# Three New Species, Two New Records and Four New Collections of Tubeufiaceae from Thailand and China

**DOI:** 10.3390/jof8020206

**Published:** 2022-02-20

**Authors:** Xingguo Tian, Samantha C. Karunarathna, Rongju Xu, Yongzhong Lu, Nakarin Suwannarach, Ausana Mapook, Danfeng Bao, Jianchu Xu, Saowaluck Tibpromma

**Affiliations:** 1 Center for Yunnan Plateau Biological Resources Protection and Utilization, College of Biological Resource and Food Engineering, Qujing Normal University, Qujing 655011, China; 6271105511@lamduan.mfu.ac.th (X.T.); samantha@mail.kib.ac.cn (S.C.K.); 2 Centre for Mountain Futures, Kunming Institute of Botany, Kunming 650201, China; 3 School of Food and Pharmaceutical Engineering, Guizhou Institute of Technology, Guiyang 550003, China; yzlu@git.edu.cn; 4 Center of Excellence in Fungal Research, Mae Fah Luang University, Chiang Rai 57100, Thailand; xurongju1005@outlook.com (R.X.); phung.ausana@gmail.com (A.M.); baodan_feng@cmu.ac.th (D.B.); 5 School of Science, Mae Fah Luang University, Chiang Rai 57100, Thailand; 6 CIFOR-ICRAF China Program, World Agroforestry (ICRAF), Kunming 650201, China; 7 Yunnan Key Laboratory of Fungal Diversity and Green Development, Kunming Institute of Botany, Kunming 650201, China; 8 Research Center of Microbial Diversity and Sustainable Utilization, Faculty of Science, Chiang Mai University, Chiang Mai 50200, Thailand; suwan_461@hotmail.com; 9 College of Agriculture & Biological Science, Dali University, Dali 671003, China

**Keywords:** three new species, two new records, asexual morph, phylogeny, taxonomy, *Tubeufia laxispora*

## Abstract

Tubeufiaceae, a cosmopolitan family with a worldwide distribution, is mostly reported as saprobic on decaying woody materials from both aquatic and terrestrial habitats. The family is commonly found as helicosporous hyphomycetes, while some are chlamydosporous and phragmosporous. In this study, thirteen helicosporous hyphomycetes were collected from Thailand and China. The phylogenetic analyses of combined ITS, LSU, TEF1-α, and RPB2 sequence data placed them in *Dematiohelicomyces*, *Helicoma*, *Helicotruncatum*, *Neohelicosporium*, *Parahelicomyces*, and *Tubeufia* within Tubeufiaceae. Three new species, *Tubeufia cocois*, *Parahelicomyces chiangmaiensis*, and *Neohelicosporium bambusicola*, one new host record, *Tubeufia laxispora*, and one new geographic record, *T. longihelicospora*, are introduced based on both morphological characteristics and phylogenetic analyses. In addition, *Dematiohelicomyces helicosporus*, *Helicoma guttulatum*, *Helicotruncatum palmigenum*, and *Tubeufia cylindrothecia* are described with detailed descriptions and color photo plates.

## 1. Introduction

The order Tubeufiales was introduced by Boonmee et al. [1] to accommodate a single family Tubeufiaceae based on phylogenic evidence. Later, the other two families, Bezerromycetaceae and Wiesneriomycetaceae, were accepted into Tubeufiales by Liu et al. [2] based on phylogenetic analysis and divergence time estimates. Tubeufiales currently includes three families viz. Bezerromycetaceae, Tubeufiaceae, and Wiesneriomycetaceae, of which Bezerromycetaceae and Wiesneriomycetaceae are known for only sexual morphs, while Tubeufiaceae has been reported to have both sexual and asexual morphs, and the asexual morphs are mostly found as helicosporous hyphomycetes [2,3,4].

Tubeufiaceae was introduced by Barr [5] with *Tubeufia* as the generic type. The sexual morph of Tubeufiaceae is characterized by superficial ascomata, pseudoparaphysate hamathecium, and multiseptate, which are hyaline to pale brown cylindrical ascospores [5,6,7,8,9,10], while the asexual morph is hyphomycetous, mostly helicosporous, and some are chlamydosporous and phragmosporous [3,11,12]. Tubeufiaceae was recently revised by Lu et al. [3], providing an updated multi-gene phylogenetic tree for Tubeufiales with 13 new genera in this family. Currently, the family comprises 46 genera viz: *Acanthohelicospora*, *Acanthophiobolus*, *Acanthostigma*, *Acanthostigmina*, *Acanthotubeufia*, *Aquaphila*, *Artocarpomyces*, *Berkleasmium*, *Bifrontia*, *Boerlagiomyces*, *Camporesiomyces*, *Chaetosphaerulina*, *Chlamydotubeufia*, *Dematiohelicoma*, *Dematiohelicomyces*, *Dematiohelicosporum*, *Dematiotubeufia*, *Dictyospora*, *Discotubeufia*, *Helicangiospora*, *Helicoarctatus*, *Helicodochium*, *Helicohyalinum*, *Helicoma*, *Helicomyces*, *Helicosporium*, *Helicotruncatum*, *Helicotubeufia*, *Kamalomyces*, *Kevinhydea*, *Manoharachariella*, *Muripulchra*, *Neoacanthostigma*, *Neochlamydotubeufia*, *Neohelicoma*, *Neohelicomyces*, *Neohelicosporium*, *Neotubeufia*, *Pleurohelicosporium*, *Podonectria*, *Pseudohelicomyces*, *Pseudohelicoon*, *Tamhinispora*, *Thaxteriella*, *Thaxteriellopsis*, and *Tubeufia* [3,4]. Tubeufiaceae is cosmopolitan with a worldwide distribution in both tropical and temperate regions [1,3,5,12].

Previously, the taxonomic placement of Tubeufiaceae was uncertain, thus it has been discussed by several mycologists. First, Barr [5] accommodated Tubeufiaceae in Pleosporales based on the generic type *Tubeufia* and later it was followed by various mycologists [6,7,13,14,15]. Eriksson and Winka [16], and Eriksson [17,18] preferred to accommodate Tubeufiaceae within the Dothideales, while Eriksson [19], as well as Lumbsch and Huhndorf [20], preferred to accommodate Tubeufiaceae within Dothideomycetes and Chaetothyriomycetes incertae sedis. Based on 28S rDNA sequence data, Kodsueb et al. [21] preferred to keep Tubeufiaceae in Pleosporales (as the natural placement), which also follows the ordinal circumscription of Barr [6,7], Sivanesan [13], Rossman, Crane et al. [14], and Kirk et al. [15]. Species of Tubeufiaceae are commonly reported as saprobes on woody substrates or submerged decaying wood in terrestrial and aquatic habitats [1,2,3,10,12,22]. Several studies reported that Tubeufiaceae species are able to produce active secondary metabolites, which have potential anti-fungal, anti-bacterial, anti-diabetic, and anti-cancer properties [3,23,24,25]. Several Tubeufiaceae studies have been carried out based on descriptions, illustrations, and phylogenetic evidence in Asia, especially in China, India, Japan, and Thailand [1,2,3,4,12,26].

In this study, thirteen helicosporous hyphomycetes were collected from Thailand and China. Phylogenetic analyses of combined ITS, LSU, TEF1-α, and RPB2 sequence data place them in *Dematiohelicomyces*, *Helicoma*, *Helicotruncatum*, *Neohelicosporium*, *Parahelicomyces*, and *Tubeufia*. Three new species, *Tubeufia cocois*, *Parahelicomyces chiangmaiensis*, and *Neohelicosporium bambusicola* are introduced with morphological and phylogenic evidence. One new host record, *Tubeufia laxispora*, from *Cocos nucifera* in Thailand, as well as one new geographic record, *T. longihelicospora*, in China, are introduced. In addition, four known species, *Dematiohelicomyces helicosporus*, *Helicoma guttulatum*, *Helicotruncatum palmigenum*, and *Tubeufia cylindrothecia* are also described. Full descriptions, color photographs, and a phylogenetic tree to show the placement of nine taxa are provided.

## 2. Materials and Methods

### 2.1. Sample Collection, Isolation, and Specimen Examination

Decaying wood, leaves, and culms were collected from Chiang Rai and Chiang Mai Provinces of Thailand from May 2020 to March 2021 and Yunnan Province of China in September 2021. Specimens were brought to a mycology laboratory for observation. Tian et al. [27,28] and Senanayake et al. [29] were followed for the morphological study and single spore isolation. Morphological characteristics were examined under a stereomicroscope (Motic SMZ-171, Wetzlar, Germany). Conidiomata were observed and photographed using a Nikon ECLIPSE Ni-U compound microscope connected with a Nikon camera series DS-Ri2. Germinating conidia were transferred aseptically to a potato dextrose agar (PDA) medium, incubated at 28 °C for 2–4 weeks, and the morphological characteristics of cultures were recorded.

Herbarium specimens were deposited at the herbarium of the Mae Fah Luang University (MFLU) and Kunming Institute of Botany (HKAS), while the living cultures were deposited at Mae Fah Luang University Culture Collection (MFLUCC) and Kunming Institute of Botany Culture Collection (KUMCC). Faces of Fungi and Index Fungorum numbers were registered as outlined in Index Fungorum [30] and Jayasiri et al. [31].

### 2.2. DNA Extraction, PCR Amplification, and Sequencing

Genomic DNA was extracted from two-week-old living pure cultures grown on PDA using the Biospin Fungus Genomic DNA extraction Kit (BioFlux, Kun Ming, P.R. China) following the manufacturer’s protocol. DNA was subjected to PCR amplification to amplify the genes ITS, LSU TEF1-α, and RPB2, while internal transcribed spacer (ITS) with the primer pair of ITS4/ITS5 [32], the partial large subunit nuclear rDNA (LSU) with the primer pair of LR0R/LR5 [33], the translation elongation factor 1-alpha gene (TEF1-α) with the primer pair of EF1–983F/EF1–2218R [34], and RNA polymerase II second largest subunit (RPB2) with the primer pair of RPB2–5f/7cR [35]. The PCR was carried out using the method described by Tian et al. [28]. ITS, LSU, and TEF1-α amplification reactions were set using the method described by Cai et al. [36] and Lu et al. [37]. RPB2 amplification reaction was set using the method described by Lu et al. [3]. PCR products were checked and purified in 1% agarose gels and were sequenced at TsingKe Biological Technology (Kunming) Co., China.

### 2.3. Phylogenetic Analyses

The raw sequences (ITS, LSU, TEF1-α, and RPB2) were spliced using SeqMan and subjected to BLAST in GenBank to find closely related taxa. Sequences of four genes downloaded from NCBI GenBank are listed in Table 1. A single gene sequence alignment was generated with MAFFT v.7.110 online application [38,39] and trimmed using trimAl v 1.2 with the ‘gappyout’ option [40]. Multiple genes were concatenated by Sequence Matrix. Multigene phylogenetic analyses of the concatenated genes were reconstructed from maximum likelihood (ML) and Bayesian inference (BI) analyses. Maximum likelihood was performed using the online RAxML-HPC on XSEDE tool on CIPRES under the GTRGAMMA substitution model and 1000 bootstrap replicates [38,41,42]. Bayesian inference analysis was performed using the MrBayes on XSEDE tool on CIPRES [42]. The best-fit models were selected as GTR+I+G for ITS, LSU, TEF1-α, and RPB2 for the Bayesian posterior probability analysis. Two parallel runs were conducted using the default settings, six simultaneous Markov chains were run for 50,000,000 generations, and trees were sampled every 500th generation. The alignment generated in this study was submitted to TreeBASE (https://treebase.org/treebase-web/home.html, accessed on 10 January 2022) under the submission number ID29068. Trees were visualized with FigTree v1.4.4, and layouts were carried out with Adobe Illustrator CS5 v. 16.0.0.

## 3. Results

### 3.1. Phylogenetic Analyses

**Figure 1:**
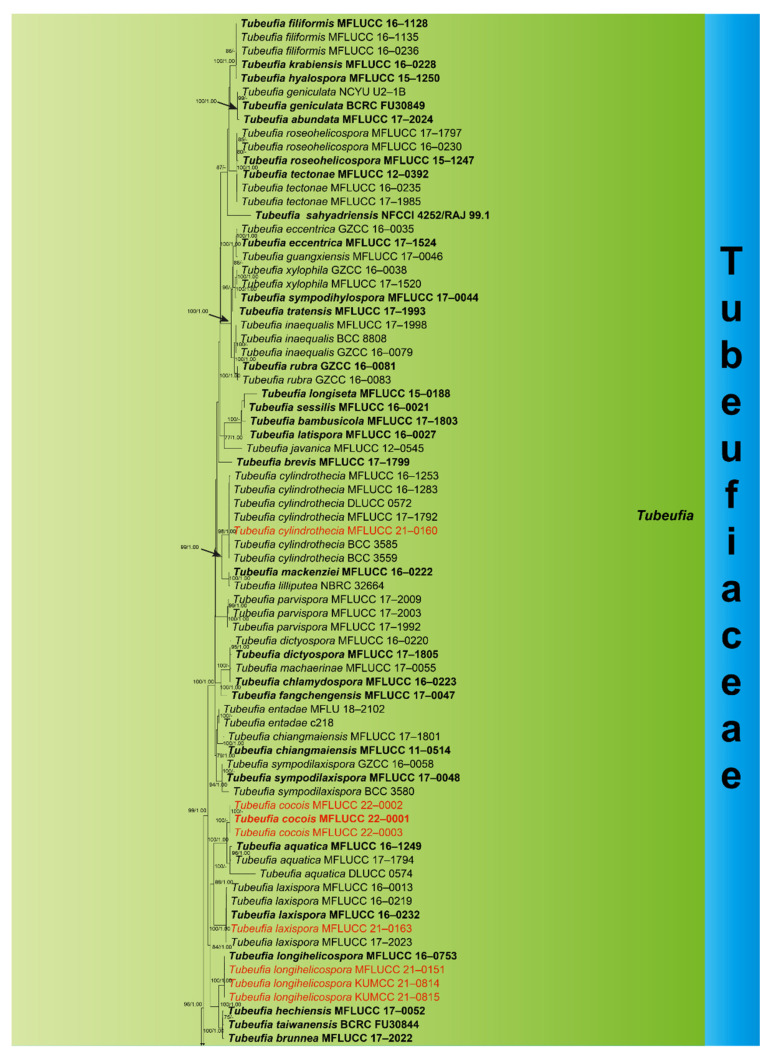

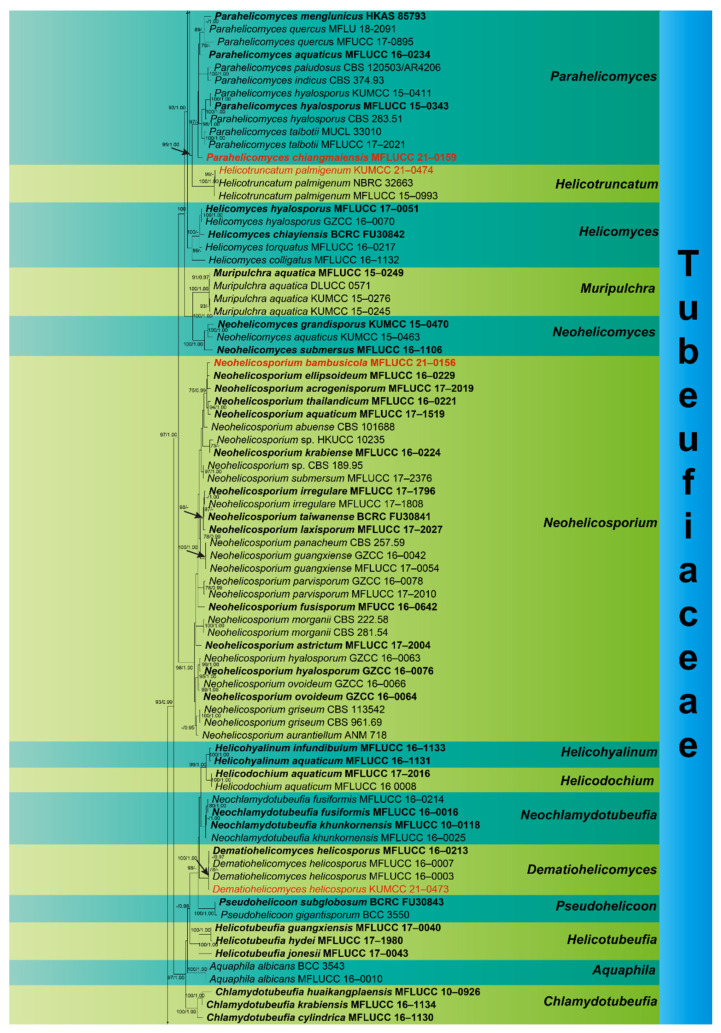

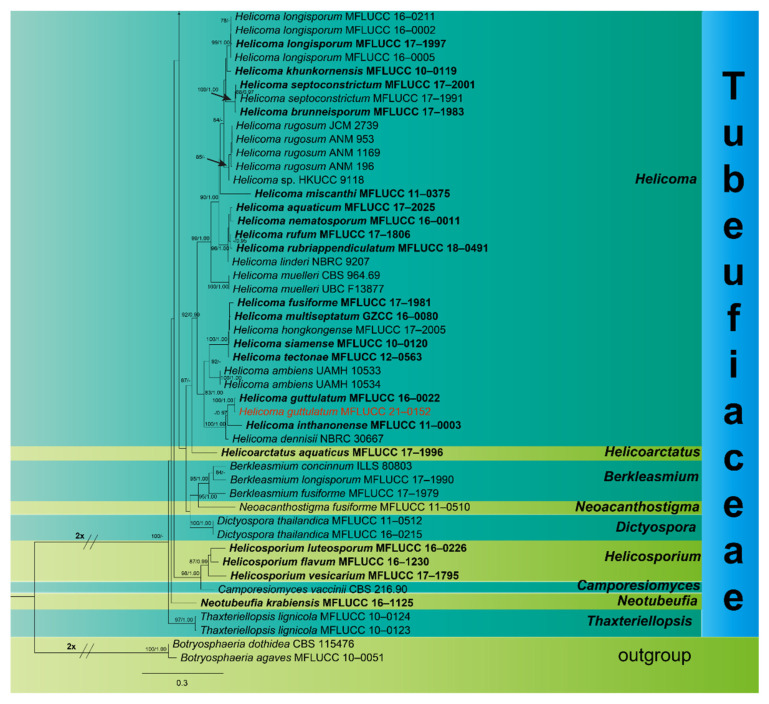
Phylogenetic tree generated from a maximum likelihood analysis based on a concatenated alignment of ITS, LSU, TEF1-α, and RPB2 sequences data in Tubeufiaceae. The tree is rooted with *Botryosphaeria dothidea* (CBS 115476) and *B. agaves* (MFLUCC 10–0051). Bootstrap support values equal to or higher than 75% ML (**left**) or posterior probability values equal to or higher than 0.95 Bayesian PP (**right**) are indicated on the nodes. Newly generated sequences are in red. Ex-type strains are in black/red bold.

The combined ITS, LSU, TEF1-α, and RPB2 dataset comprised thirteen newly sequenced strains, with *Botryosphaeria dothidea* (CBS 115476) and *B. agaves* (MFLUCC 10–0051) as outgroup taxa. Multiple genes were concatenated, which comprised 3425 nucleotide characters, including gaps (ITS: 1–606 bp, LSU: 607–1471 bp, RPB2: 1472–2514 bp, TEF1-α: 2515–3425 bp). The RAxML analysis of the combined dataset yielded the best-scoring tree (Figure 1) with a final ML optimization likelihood value of −50085.613741. The matrix had 1671 distinct alignment patterns, with 26.72% undetermined characters or gaps. Estimated base frequencies were as follows: A = 0.244864, C = 0.251768, G = 0.258885, T = 0.244483; substitution rates AC = 1.177463, AG = 5.880242, AT = 2.143397, CG = 0.867985, CT = 9.022451, GT = 1.000000; gamma distribution shape parameter α = 0.224491.

Phylogenetic analyses showed that our thirteen collections were placed within Tubeufiaceae viz: *Dematiohelicomyces*, *Helicoma*, *Helicotruncatum*, *Neohelicosporium*, *Parahelicomyces*, and *Tubeufia*. Eight collections clustered within *Tubeufia*; the new strain *Tubeufia cylindrothecia* (MFLUCC 21–0160) was nested with six strains of *T. cylindrothecia* with strong bootstrap support (98% ML/1.00 PP). Three strains of *Tubeufia cocois* (MFLUCC 22–0001, MFLUCC 22–0002, and MFLUCC 22–0003) clustered together and formed a branch at the basal clades of *T. aquatica* with strong bootstrap support (100% ML/1.00 PP). The new strain *T. laxispora* (MFLUCC 21–0163) nested with four strains of *T. laxispora* with strong bootstrap support (100% ML/1.00 PP), and *T. longihelicospora* (MFLUCC 21–0151, KUMCC 21–0478, and KUMCC 21–0479) clustered together within the same clade as *T. longihelicospora* (MFLUCC 16–0753). *Parahelicomyces chiangmaiense* (MFLUCC 21–0159) formed a single branch at the basal clades of *Parahelicomyces* members with strong support (97% ML). Newly obtained strain *Helicotruncatum palmigenum* (KUMCC 21–0474) nested with two strains of *H. palmigenum* (NMRC 32,663 and MFLUCC 15–0093) strong bootstrap support (100% ML/1.00 PP). *Neohelicosporium bambusicola* (MFLUCC 21–0160) was placed as a sister taxon to *N. ellipsoideum* (MFLUCC 16–0229) and *N. acrogenisporum* (MFLUCC 17–2019). New strain *Dematiohelicomyces helicosporus* (KUMCC 21–0473) clustered with three strains of *Dematiohelicomyces helicosporus* with strong bootstrap support (100% ML/1.00 PP), and *Helicoma guttulatum* (MFLUCC 21–0152) clustered with its ex-type strain of *H. guttulatum* (MFLUCC 16–0022) with high support (100% ML/1.00 PP).

### 3.2. Taxonomy

#### 3.2.1. *Dematiohelicomyces* Y.Z. Lu, Boonmee, and K.D. Hyde, Fungal Diversity 92: 159 (2018)

Index Fungorum, IF 554824; Facesoffungi number, FoF 04701

Type species: *Dematiohelicomyces helicosporus* (Boonmee, Y.Z. Lu, and K.D. Hyde) Y.Z. Lu

The monotypic genus *Dematiohelicomyces* was introduced by Lu et al. [3], with *D. helicosporus* as the type species based on morphology and phylogeny. *Dematiohelicomyces* are saprobic on submerged decaying wood in a freshwater stream in Thailand. *Dematiohelicomyces* is characterized by short conidiophores that are brown, 0–3-septate, and helicoid conidia, with a spathulate basal end cell. In this paper, *Dematiohelicomyces helicosporus* was collected from submerged decaying wood in a freshwater river in Thailand.

**Figure 2:**
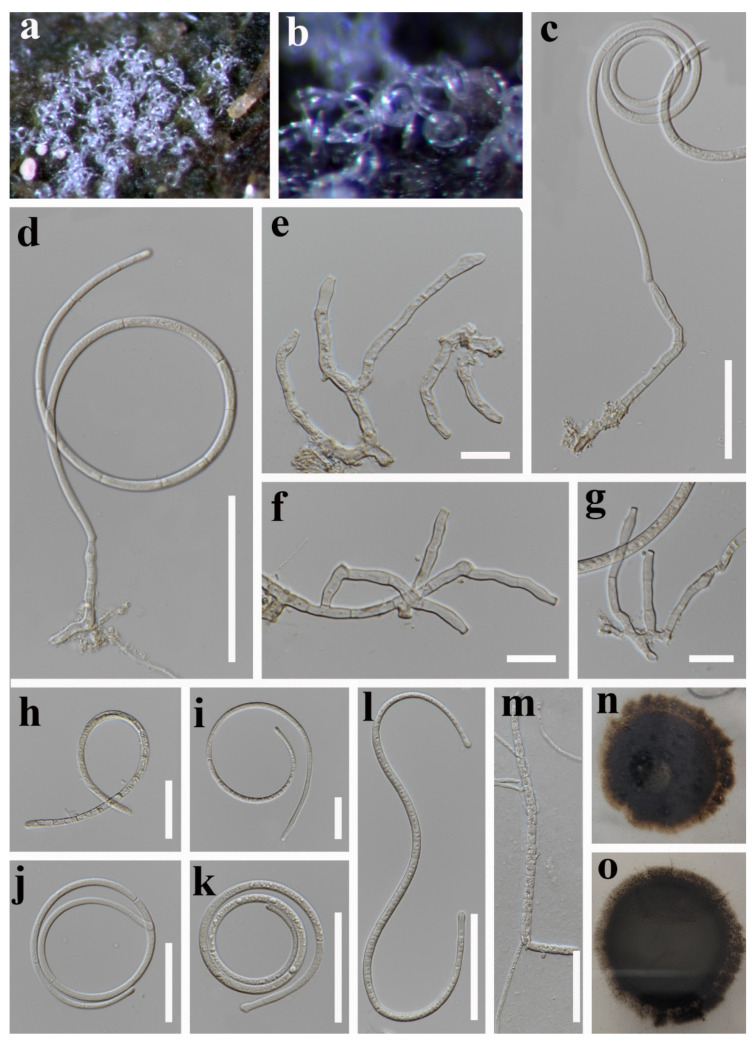
***Dematiohelicomyces helicosporus*** (MFLU 21–0184). (**a**,**b**) Colony on decaying wood; (**c**,**d**) conidiophores and conidia; (**e**–**g**) conidiophores and conidiogenous cells; (**h**–**l**) conidia; (**m**) germinated conidium; (**n**,**o**) colony cultures on PDA (observe and reverse). **Scale bars**: (**j**) = 80 µm, (**d**) = 60 µm, (**c**,**h**,**i**,**k**–**m**) = 40 µm, (**e**–**g**) = 20 µm.

***Dematiohelicomyces helicosporus*** (Boonmee, Y.Z. Lu, and K.D. Hyde) Y.Z. Lu, Fungal Diversity 92: 159 (2018) (Figure 2).

≡*Chlamydotubeufia helicospora* Boonmee, Y.Z. Lu, and K.D. Hyde, Fungal Diversity 80: 123 (2016)

Index Fungorum, IF 554825; Facesoffungi number, FoF 04702

*Saprobic* on submerged decaying wood in a freshwater stream. **Sexual morph** Undetermined. **Asexual morph** Hyphomycetous, helicosporous. *Colonies* are superficial, effuse, gregarious, white, and shiny. *Mycelium* is mostly immersed, composed of branched, septate hyphae, brown, with masses of glistening, crowded conidia. *Conidiophores* (16.5–)30–65.5(–80.5) × 4–5 μm ($\bar{x}$ = 47 × 4.5 μm, n = 25) are macronematous, erect, cylindrical, branched, 0–4-septate, hyaline to pale brown, arising as lateral branches from creeping hyphae, and smooth-walled. *Conidiogenous cells* (9–)14–24.5(–30) × 4–5 μm ($\bar{x}$ = 19.5 × 4.5 μm, n = 30) are holoblastic, monoblastic, integrated, terminal, cylindrical, truncate at the apex after conidial secession, hyaline, and smooth-walled. *Conidia* are solitary, acrogenous, helicoid, rounded at tip, with the basal cells broadly spathulate and bearing a flattened attachment scar, guttulate, hyaline, with a (59–)80–139(–158) μm ($\bar{x}$ = 110 μm, n = 25) diam. and conidial filament 5–6.5 μm ($\bar{x}$ = 5.5 μm, n = 25) wide in the broadest part, tapering towards the ends, 328–482.5 μm ($\bar{x}$ = 405.5 μm, n = 25) long, multi-septate, coiled 1–2 ½times, tightly to loosely coiled in water, smooth-walled, and contain granules.

Culture characteristics: conidia germinating on PDA within 12 h; colonies growing on PDA, reaching 20 mm in 2 weeks at 28 °C, circular, with a flat surface, edge entire, pale brown to dark brown in PDA medium; mycelium partially immersed, branched, multi-septate, hyaline to pale brown, smooth.

Material examined: Thailand, Chiang Rai Province, Mae Fah Luang University, on submerged decaying wood, 22 May 2020, R. J. Xu, MD38 (MFLU 21–0184), living culture, KUMCC 21–0473.

Notes: There are some differences between our new isolate (KUMCC 21–0473) and *D. helicosporus* morphologically, such as conidiogenous cells in our new isolate are monoblastic, while in *D. helicosporus* (MFLUCC 16–0003), they are mono- to polyblastic. In addition, the new isolate (KUMCC 21–0473) differs from *D. helicosporus* (MFLUCC 16–0003) in having larger (80–139 vs. 70–100 μm) and shorter conidial filaments (328–482.5 vs. 400–600 μm) [3,43]. However, our phylogenetic results show that the new isolate *D. helicosporus* (KUMCC 21–0473) clusters with three strains of *D. helicosporus* (MFLUCC 16–0003, MFLUCC 16–0007, and MFLUCC 16–0213) with high statistical supports (100% ML/1.00 PP, Figure 1). Therefore, we identify our new isolate as *Dematiohelicomyces helicosporus*. *Chlamydotubeufia helicospora* was collected on decaying wood in a flowing freshwater stream in Uttaradit Province, Thailand [43], and later, based on morphology and phylogeny, Lu et al. [3] synonymized this taxon under *Dematiohelicomyces helicosporus*. In this study, our new isolate was also collected from a submerged decaying wood in Chiang Rai Province, Thailand, which is a little far from the original collection location, meaning that this species still prefers similar environmental conditions.

#### 3.2.2. *Helicoma* Corda, Icon. fung. (Prague) 1: 15 (1837)

Index Fungorum: IF 8473

Type species: *Helicoma muelleri* Corda, Icon.

*Helicoma* was introduced by Corda [44], with *H. muelleri* as a type species. Two types of asexual morphs have been observed in *Helicoma*: the first asexual morphs are characterized by conidiogenous cells that are cylindrical, with denticles, intercalary, arising laterally from the lower portion of conidiophores, and conidia are pleurogenous, tapering towards the apex and rounded at the tip, helicoid, hygroscopic, and become loosely coiled in water [12]. Another asexual morph is characterized by conidia that are acrogenous or acropleurogenous, helicoid, circinate, dry, tapering towards the apex, truncating at the base, coiled 1¼–¾ times, and not becoming loose in the water. There are 97 records listed in Index Fungorum (2021), however, most of them are lacking sequence data in GenBank. The last treatment of *Helicoma* was provided by Lu et al. [3], and they accepted 57 species within the genus while introducing 10 new species and 11 new combinations. In this study, the new isolate is identified as *H. guttulatum* based on both phylogenetic analysis and morphological characteristics.

**Figure 3:**
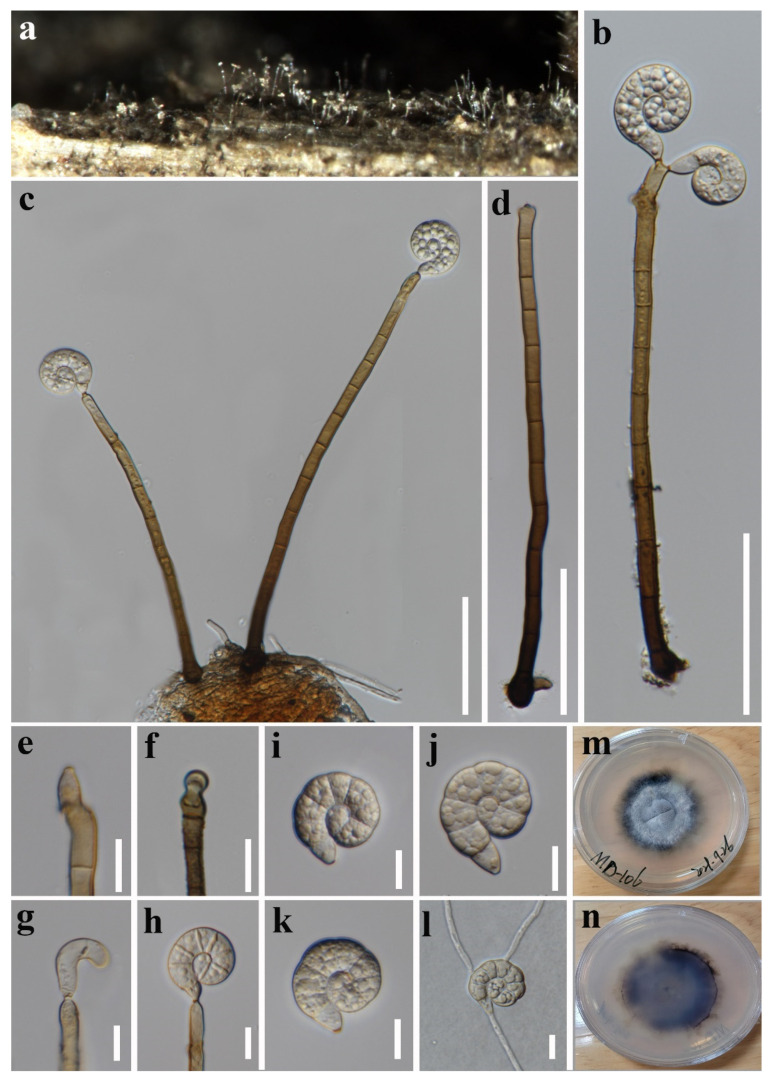
*Helicoma guttulatum* (MFLU 21–0183). (**a**) Colony on decaying wood; (**b**,**c**) conidiophores and conidia; (**d**) conidiophore; (**e**,**f**) tip of conidiogenous cells; (**g**,**h**) conidiogenous cells and conidia; (**i**–**k**) conidia; (**l**) germinated conidium**;** (**m**,**n**) colony cultures on PDA (observe and reverse). **Scale bars**: (**b**) = 40 µm, (**c**,**d**) = 60 µm, (**e**–**l**) = 20 µm.

***Helicoma guttulatum*** Y.Z. Lu, Boonmee, and K.D. Hyde, Fungal Diversity 80: 125 (2016) (Figure 3).

Index Fungorum, IF 552218; Facesoffungi, FoF 02358

*Saprobic* on submerged decaying wood in a freshwater stream. **Sexual morph** Undetermined. **Asexual morph** Hyphomycetous, helicosporous. *Colonies* are superficial, effuse, gregarious, brown to dark brown. *Mycelium* is mostly immersed, partly superficial, composed of branched, septate, brown hyphae. *Conidiophores* (65–)93–156.5 × 4.5–6 μm ($\bar{x}$ = 125 × 5 μm, n = 20) are macronematous, mononematous, cylindrical, septate, erect, unbranched, pale brown to brown at the apex and dark brown at the base, and smooth-walled. *Conidiogenous cells* (9–)12–24(–30.5) × 4–5.5 μm ($\bar{x}$ = 18 × 4.5 μm, n = 20) are holoblastic, mono- to polyblastic, integrated, terminal, cylindrical, brown, and smooth-walled. *Conidia* 22–26.5 μm ($\bar{x}$ = 24 μm, n = 25) have a diam. and conidial filament 7–8.5 μm ($\bar{x}$ = 8 μm, n = 25) wide and 49–58 μm ($\bar{x}$ = 53.5 μm, n = 25) long, are integrated, terminal, helicoid, tightly coiled 1–1½ times, guttulate, do not become loose in the water, 8-septate, straight constricted at the septa, subhyaline to yellowish, rounded at the apex, and smooth-walled.

Culture characteristics: conidia germinated on PDA within 12 h; colonies growing on PDA, reaching 25 mm in 2 weeks at 28 °C, circular, with a flat surface, edge entire, and pale brown to brown in PDA medium; mycelium were partially immersed, branched, multi-septate, hyaline to pale brown, and smooth.

Material examined: Thailand, Chiang Rai Province, Mueang, Nang Lae on submerged decaying wood, 14 August 2020, R. J. Xu, MD106 (MFLU 21–0183), living culture, MFLUCC 21–0152.

Notes: *Helicoma guttulatum* was introduced by Hyde et al. [43] on submerged decaying wood from a freshwater stream in Thailand. In our phylogenetic analyses, the newly obtained isolate (MFLUCC 21–0152) clustered with the ex-type strain of *H. guttulatum* (MFLUCC 16–0022) with high statistical support (100% ML/1.00 PP, Figure 1). Morphologically, the new isolate was indistinguishable from the holotype of *H. guttulatum* [43]. Therefore, we identify the new isolate as *Helicoma guttulatum* based on morphological and phylogenetic data.

#### 3.2.3. *Helicotruncatum* Y.Z. Lu, J.C. Kang, and K.D. Hyde, Fungal Diversity 92: 220 (2018)

Index Fungorum, IF 554859; Facesoffungi number, FoF 04,730

Type species: *Helicotruncatum palmigenum* (Penz. and Sacc.) Y.Z. Lu and K.D. Hyde

The monotypic genus *Helicotruncatum* was established by Lu et al. [3] with *H. palmigenum* as the type species, and it is the only species accepted in the genus [3,12]. *Helicotruncatum palmigenum* was originally placed in *Helicoma,* based on morphological characters [12,45,46]. Phylogenetic analysis of Lu et al. [3] showed that *H. palmigenum* formed an independent lineage and was distant from *Helicoma*. Morphologically, *H. palmigenum* can be distinguished from other helicosporous hyphomycetes by the distinctively thickened lateral cell wall of the conidiophore and basal cell of the conidium. Thus, Lu et al. [3] introduced a new genus, *Helicotruncatum,* to accommodate *H. palmigenum* based on both phylogeny and morphology. In this paper, *Helicotruncatum palmigenum* was collected from dead *Cocos nucifera* leaves in Thailand.

**Figure 4:**
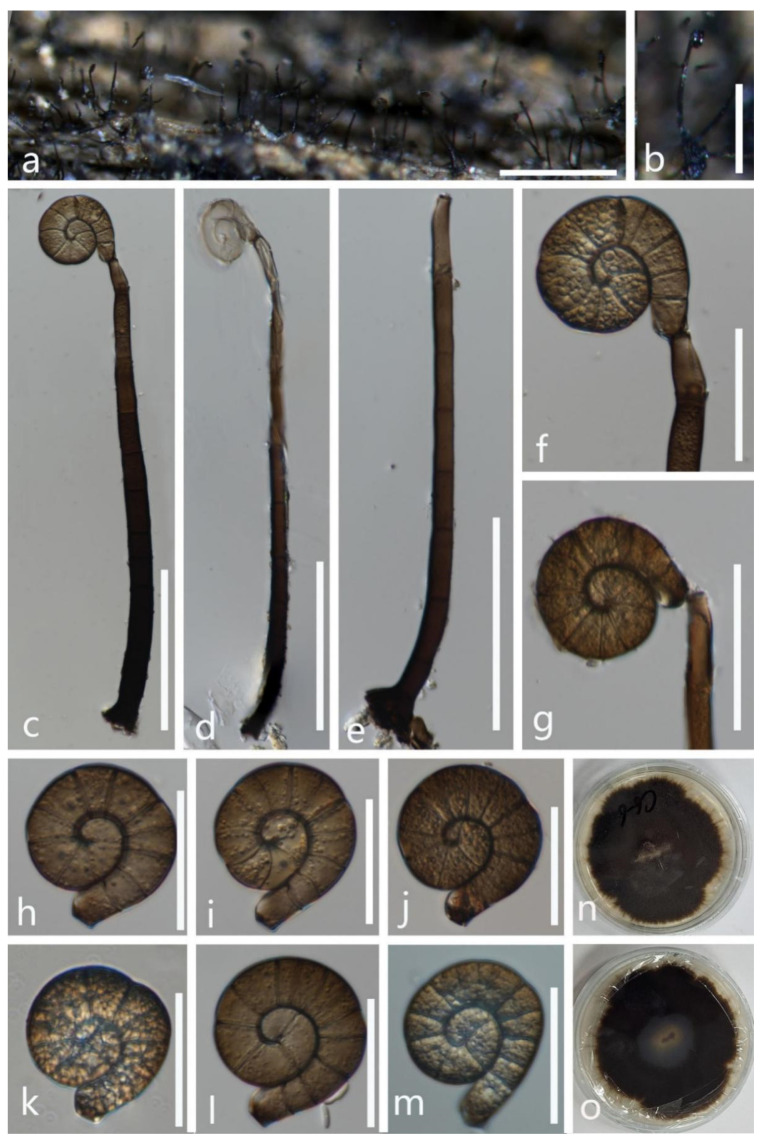
*Helicotruncatum palmigenum* (MFLU 21–0185). (**a**,**b**) Colony on decaying leaves; (**c**,**d**) conidiophores, conidiogenous cells, and conidia; (**e**) conidiophores and conidiogenous cells; (**f**,**g**) conidiogenous cells and conidia; (**h**–**m**) conidia; (**n**,**o**) colony cultures on PDA (observe and reverse). **Scale bars**: (**a**) = 500 µm, (**b**) = 200 µm, (**c**–**e**) = 80 µm, (**f**–**m**) = 30 µm.

***Helicotruncatum palmigenum*** (Penz. and Sacc.) Y.Z. Lu and K.D. Hyde, Fungal Diversity 92: 220 (2018) (Figure 4).

≡*Helicosporium intermedium* var. *palmigenum* Penz. and Sacc., Malpighia 15(7–9): 249 (1902)

≡*Helicoma palmigenum* (Penz. and Sacc.) Linder, Ann. Mo. bot. Gdn 16: 306 (1929)

=*Helicoma westonii* Linder [as ‘westoni’], Ann. Mo. bot. Gdn 18: 12 (1931)

Index Fungorum: IF 554860; Facesoffungi number: FoF 04800

*Saprobic* on dead leaves of *Cocos nucifera*. **Sexual morph**: Undetermined. **Asexual morph** Hyphomycetous, helicosporous. *Colonies* on the substratum are superficial, effuse, gregarious, and velvety black. *Mycelium* is composed of brown, septate hyphae. *Conidiophores* 165.5–283.5 × 6.5–10 µm ($\bar{x}$ = 224.5 × 8.5 µm, n =10) are macronematous, mononematous, cylindrical, stout, septate, erect, unbranched, pale brown to subhyaline at the apex and dark brown at the base, and smooth-walled. *Conidiogenous cells* 22–34.5 × 6–7.5 µm ($\bar{x}$ = 28 × 6.5 µm, n =15) are holoblastic, monoblastic, integrated, determinate, cylindrical, terminal, smooth-walled, and truncate at the apex after conidial secession. *Conidia* 32–44 μm ($\bar{x}$ = 38 μm, n = 20) and conidial filament are 10.5–15 μm ($\bar{x}$ = 12.5 μm, n = 20) wide, 82–108.5 μm ($\bar{x}$ = 95.5 μm, n = 20) long, solitary, terminal, smooth-walled, helicoid, coiled 1½–2¾ times, do not become loose in the water, septate, not constricted at septa, dilute fuliginously, and the basal cell is truncated with thickened lateral walls.

Culture characteristics: conidia germinating on PDA within 12 h; colonies reaching 40 mm in 2 weeks at 28 °C, irregular, dark brown from above and pale brown from below; mycelium are slow-growing, thin, and effuse brownish grey.

Material examined: Thailand, Chiang Rai Province, on decaying leaves of *Cocos nucifera*, 16 January 2021, X. G. Tian, C6–6, (MFLU 21–0185), living culture, KUMCC 21–0474.

Notes: In the phylogenetic analyses, our new collection KUMCC 21–0474 clusters with two strains of *H. palmigenum* (NBRC 32663, MFLUCC 15–0993) with high statistical supports (100% ML/1.00 PP, Figure 1). Morphologically, our new isolate is almost identical to *H. palmigenum* except for the size of the conidiogenous cells (22–34.5 vs. 17–25 µm long) and the conidia (82–108.5 vs. 50–60 µm long). The nucleotide comparisons show 2 bp and 1 bp of ITS and LSU differences between the new isolate (KUMCC 21–0474) and *H. palmigenum* (NBRC 32663)**.** Thus, we identify the new isolate as *H. palmigenum* based on both phylogenetic analyses and morphological characteristics.

*Helicotruncatum palmigenum* was introduced as *Helicoma palmigenum* by Linder [45] on decaying petioles of palms that were collected from Australia, Brazil, China, Indonesia, Japan, Mexico, New Guinea, Seychelles, Thailand, Trinidad, and the USA [3,45,46]. In addition, *Helicotruncatum palmigenum* has been reported on leaves and husks of *Cocos nucifera* [34]. Our new isolate was also collected on dead leaves of *Cocos nucifera* from Thailand.

#### 3.2.4. *Neohelicosporium* Y.Z. Lu, J.C. Kang, and K.D. Hyde, Mycological Progress 17: 637 (2017)

Index Fungorum: IF 822045

Type species: *Neohelicosporium parvisporum* Y.Z. Lu, J.C. Kang, and K.D. Hyde

*Neohelicosporium* was introduced by Lu et al. [10], with five new species. The taxonomic revision of the genus was recently provided by Lu et al. [3]; eight *Helicosporium*, two *Helicoma*, and one *Tubeufia* species were transferred to *Neohelicosporium* based on both phylogeny and morphology. The genus is characterized by superficial, ellipsoidal to subglobose, ostiolate ascomata, bitunicate, cylindrical, pedicellate asci and fusiform, straight or slightly curved, multi-septate, guttulate, hyaline, smooth-walled ascospores; macronematous, mononematous, branched or unbranched, septate, pale brown to brown conidiophores, holoblastic, mono- to polyblastic, integrated, sympodial, intercalary or terminal conidiogenous cells with denticles and solitary, acrogenous and/or acropleurogenous, helicoid, multi-septate, guttulate, hyaline to pale brown conidia. Species of the genus are saprobic on decaying woody substrates from both aquatic and terrestrial habitats [47]. In this study, the new species *Neohelicosporium bambusicola* is introduced based on both phylogenetic analysis and morphological characters.

**Figure 5:**
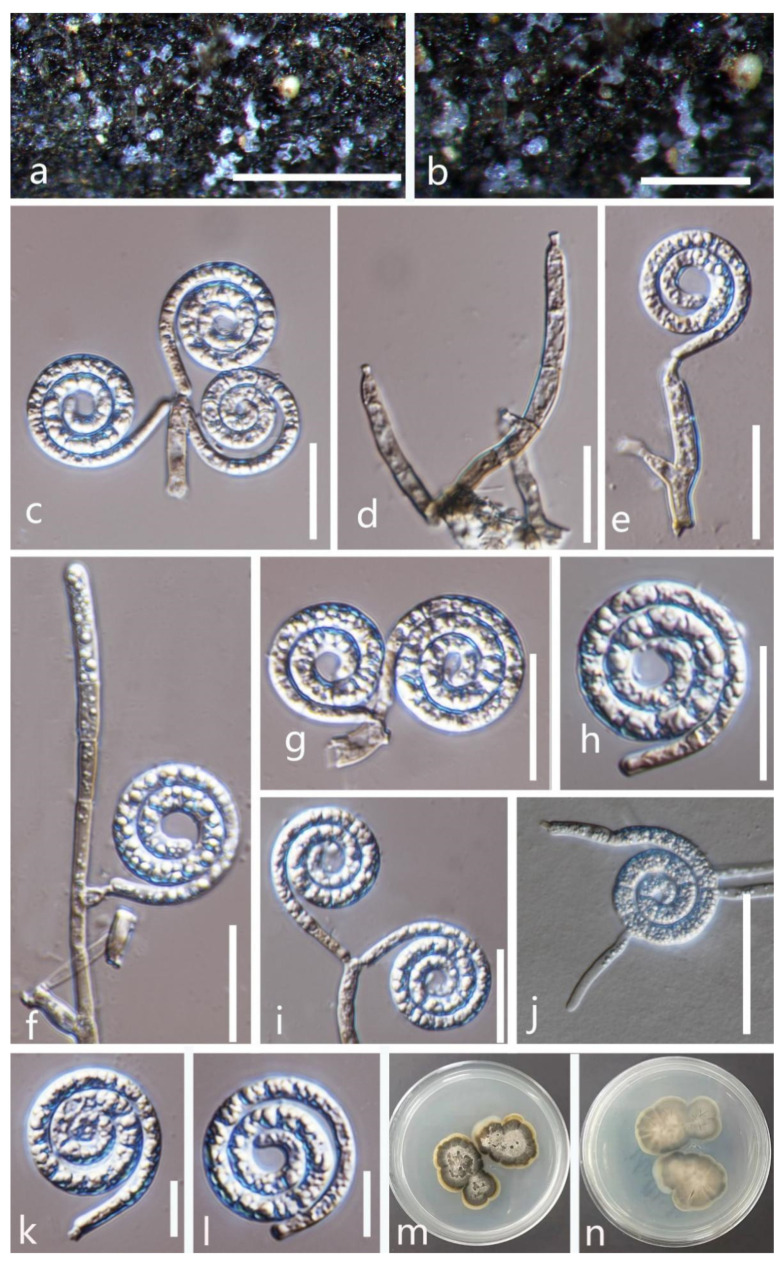
*Neohelicosporiumbambusicola* (MFLU 21–0189, holotype). (**a**,**b**) Colony on culms of bamboo; (**c**,**e**–**g**,**i**) conidiogenous cells and conidia; (**d**) conidiophores; (**h**,**k**,**l**) conidia; (**j**) germinated conidium; (**m**,**n**) colony cultures on PDA (observe and reverse). **Scale bars**: (**a**) = 500 µm, (**b**) = 200 µm, (**e**,**f**,**k**,**l**) = 20 µm, (**c**,**d**,**g**–**j**) = 10 µm.

***Neohelicosporiumbambusicola*** X.G. Tian and Tibpromma, sp. nov. (Figure 5).

Index Fungorum number, IF 555045; Facesoffungi number, FoF 10571

Etymology: Referring to the host plant bamboo, on which the fungus was collected.

*Saprobic* on terrestrial dead culms of bamboo. **Sexual morph** Undetermined. **Asexual morph** Hyphomycetous, helicosporous. *Colonies* on the substratum are superficial, effuse, and white. *Mycelium* is composed of partly immersed, hyaline to brown, septate, branched hyphae with glistening conidia. *Conidiophores* 21–76 × 3–5 µm ($\bar{x}$ = 48.5 × 4 µm, n = 10) are macronematous, mononematous, cylindrical, unbranched or branched, septate, subhyaline to brown, and smooth-walled. *Conidiogenous cells* 8.5–16 × 3–4.5 µm ($\bar{x}$ = 12 × 4 µm, n = 15) are holoblastic, mono to ployblastic, integrated, sympodial, terminal or intercalary, cylindrical, truncate at apex after conidial secession, pale brown, smooth-walled. *Conidia* are solitary, acropleurogenous, helicoid, multi-septate, guttulate, hyaline when young and become brown when mature, smooth-walled, and do not become loose in water, with a 24–30 μm ($\bar{x}$ = 27 μm, n = 20) diam. and a conidial filament 3–4.5 μm ($\bar{x}$ = 3.9 μm, n = 20) wide, 100.5–128 μm ($\bar{x}$ = 114 μm, n = 20) long, and coiled 2–2¾ times.

Culture characteristics: conidia germinated on PDA within 12 h; colonies on PDA reach 20 mm in 2 weeks at 28 °C, and are superficial, effuse, and brown; mycelium is composed of partly immersed, hyaline to brown, septate, branched, smooth hyphae.

Material examined: Thailand, Chiang Mai Province, on dead culms of bamboo, 16 December 2020, X. G. Tian, U4–10 (MFLU 21–0189 holotype), ex-type culture, MFLUCC 21–0156.

Notes: In the phylogenetic analyses, the new isolate *Neohelicosporium bambusicola* (MFLUCC 21–0156) formed a distinct lineage sister to *N. ellipsoideum* (MFLUCC 16–0229) and *N. acrogenisporum* (MFLUCC 17–2009). *Neohelicosporium bambusicola* resembles *N. ellipsoideum* and *N. acrogenisporum* in having macronematous, mononematous, unbranched or branched, septate conidiophores, holoblastic, mono- to ployblastic conidiogenous cells, and helicoid, septate conidia. However, *Neohelicosporium bambusicola* is distinct from *N. ellipsoideum* and *N. acrogenisporum* as it has shorter and narrower conidiophores (21–76 × 3–5 vs. 50–230 × 5–6 vs. 45–150 × 6–7 µm), smaller conidiogenous cells (8.5–16 × 3–4.5 vs. 15–25 × 5–6 vs. 12–15 × 4–6 µm), and narrower conidia (3–4.5 vs. 5–6 vs. 4.5–7.5 µm) [3]. Pairwise nucleotide comparisons revealed that the new isolate *Neohelicosporium bambusicola* is different from *N. ellipsoideum* (MFLUCC 16–0229) in 64/544 bp (11.76%) of the ITS, 9/814 (1.1%) of the LSU, 20/1045 bp (1.91%) of RPB2, and 15/894 bp (1.68%) of TEF1-α, while *Neohelicosporium bambusicola* is different from *N. acrogenisporum* (MFLUCC 17–2009) in 2/370 bp (0.54%) of the ITS, 6/831 (0.72%) of the LSU, 27/1045 bp (2.58%) of RPB2, and 16/894 bp (1.79%) of TEF1-α. Both phylogenetic analyses and morphological characteristics support this species as a distinct new species.

#### 3.2.5. *Parahelicomyces* Goh, in Hsieh, and Goh and Kuo, Mycological Progress 20 (2): 182 (2021)

=*Pseudohelicomyces* Y.Z. Lu, J.K. Liu, and K.D. Hyde

Index Fungorum, IF 554886; Facesoffungi number, FoF 04745

Type species: *Parahelicomyces* *talbotii* (Goos) S.Y. Hsieh, Goh, and C.H. Kuo

*Parahelicomyces* is a well-studied genus, introduced as *Pseudohelicomyces* by Lu et al. [3] with *Pseudohelicomyces* *talbotii* as the type species [3,48]. *Pseudohelicomyces* was renamed *Parahelicomyce* by Hsieh et al. [49] because Parahelicomyces was a homonym and illegitimate. Currently, seven species are accepted in the genus, and all the species have sequence data available in the GenBank database. The genus is characterized by superficial, subglobose, ellipsoidal-ovate, coriaceous, ostiolate ascomata, bitunicate, cylindrical, apically thickened and rounded asci, and fusiform, multi-septate, hyaline, smooth-walled ascospores [3], as well as macronematous, mononematous, hyaline to brown, branched, septate conidiophores, holoblastic, mono- to polyblastic, integrated, intercalary or terminal, determinate or sympodial conidiogenous cells with denticles and pleurogenous or acropleurogenous, helicoid, multi-septate, hyaline to pale brown conidia. Species of the genus *Parahelicomyces* are found from both terrestrial and freshwater habitats in China, Japan, Mexico, South Africa, and Thailand [3,50]. In this study, we introduced a new *Parahelicomyces* species from Thailand.

**Figure 6:**
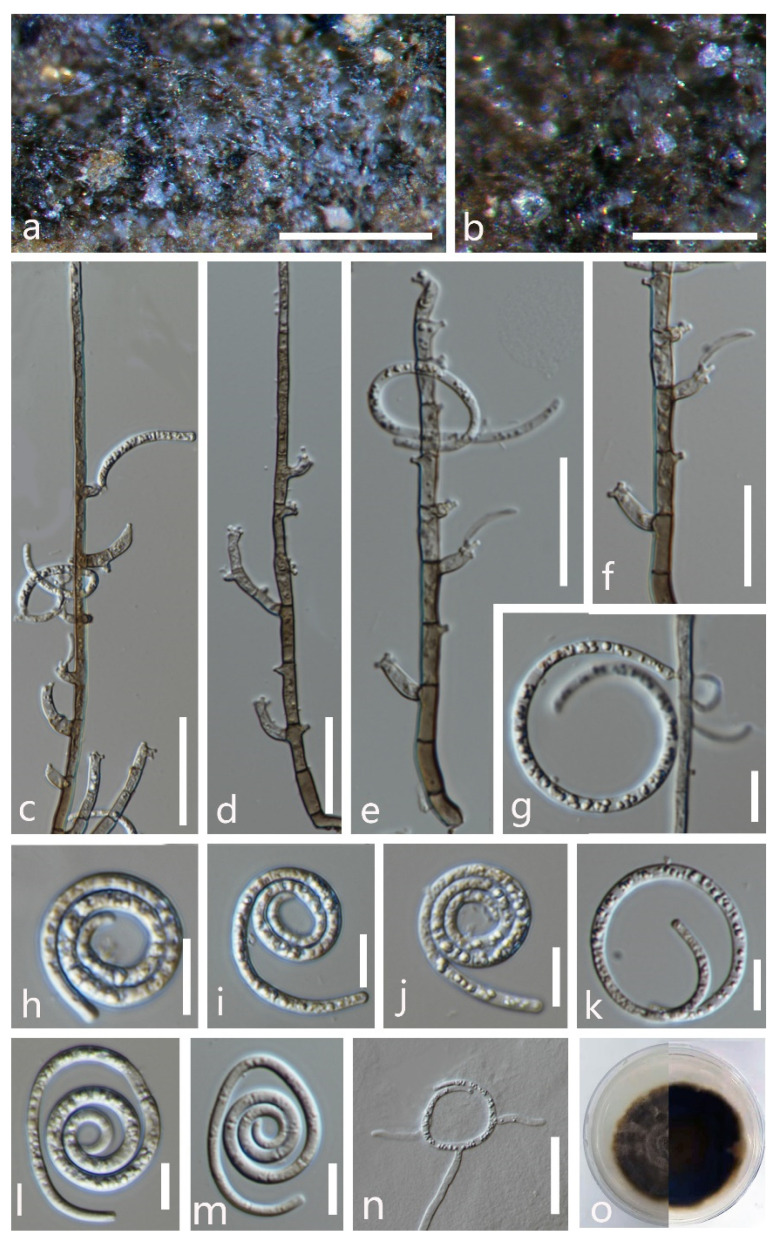
*Parahelicomyces chiangmaiensis* (MFLU 21–0188, holotype). (**a**,**b**) Colony on decaying wood; (**c**–**g**) conidiophores, conidiogenous cells, and conidia; (**h**–**m**) conidia; (**n**) germinated conidium**;** (**o**) colony cultures on PDA (observe and reverse). **Scale bars**: (**a**) = 500 µm, (**b**) = 200 µm, (**e**,**f**,**k**–**m**) = 20 µm, (**c**,**d**,**g**–**j**,**n**) = 10 µm.

***Parahelicomyceschiangmaiensis*** X.G. Tian and Tibpromma, sp. nov. (Figure 6).

Index Fungorum, IF 555060; Facesoffungi number, FoF 10570

Etymology: Referring to Chiangrai Province, Thailand, where the fungus was collected.

*Saprobic* on a terrestrial woody substrate. **Sexual morph** Undetermined. **Asexual morph** Hyphomycetous, helicosporous. *Colonies* on the substratum are superficial, effuse, gregarious, and white. *Mycelium* is composed of partly superficial, hyaline to pale brown, branched hyphae, with masses of crowded, glistening conidia. *Conidiophores* 85–180 × 2.9–3.7 µm ($\bar{x}$ = 132 × 3 µm, n =10) are macronematous, mononematous, cylindrical, pale brown to brown, paler towards the apex, straight or flexuous, branched, septate, and smooth-walled. *Conidiogenous cells* 6–10 × 2.5–3.5 µm ($\bar{x}$ = 8 × 3 µm, n =20) are holoblastic, mono- to polyblastic, integrated, sympodial, terminal or intercalary, cylindrical, with denticles, hyaline to pale brown, and smooth-walled. *Conidia* have a 21–33.5 μm ($\bar{x}$ = 27 μm, n = 20) diam. and a conidial filament 2–3 μm ($\bar{x}$ = 2.5 μm, n = 20) wide, 73–130 μm ($\bar{x}$ = 101.5 μm, n = 20) long, and coiled 1¼–3 times, and are acropleurogenous, solitary, multi-septate, helicoid, rounded at the tip, hyaline to pale brown, guttulate, tightly to loosely coiled in water, and smooth-walled.

Culture characteristics: conidia germinated on PDA within 12 h; colonies adpressed reaching 30 mm in 2 weeks at 28 °C, amd were circular, brown to dark brown, reverse brown, and slow-growing; mycelium was superficial and partially immersed, branched, septate, hyaline to pale brown, and smooth.

Material examined: Thailand, Chiang Mai Province, on the dead terrestrial woody substrate, 16 December 2020, X. G. Tian, U4–8 (MFLU 21–0188 holotype), ex-type culture, MFLUCC 21–0159.

Notes: Phylogenetic analyses of combined LSU, ITS, RPB2, and TEF1-α sequence data showed that our new isolate *Parahelicomyces chiangmaiensis* (MFLUCC 21–0159) formed an independent lineage within the genus with strong support (97% ML). *Parahelicomyces chiangmaiensis* is phylogenetically closely related to *P. talbotii* (MFLUCC 17–2021), however, *P. chiangmaiensis* can be distinguished from *P. talbotii* by the size (21–33.5 vs. 7–16 μm diam.) of conidia and the size (6–10 vs. 7–16 μm long) of conidiogenous cells. *Parahelicomyces chiangmaiensis* is morphologically closely related to *P. indicus*, however, *P. chiangmaiensis* can be distinguished from *P. indicus* by the colour (pale brown to brown vs. dark to yellowish-brown) and size (85–180 × 2.9–3.7 vs. 47–145× 3–7.5 μm) of the conidiophores [51]. Both phylogenetic analyses and morphological characteristics support *Parahelicomyces chiangmaiensis* as a distinct new species.

#### 3.2.6. *Tubeufia* Penz. and Sacc., Malpighia 11(11–12): 517 (1898)

Index Fungorum: IF 5635

Type species: *Tubeufia javanica* Penz. and Sacc., Malpighia 11(11–12): 517 (1898)

*Tubeufia*, the type genus of Tubeufiaceae, was established by Penzig and Saccardo [52]. Currently, 88 records are listed in the Index Fungorum (2021); however, most of the species are lacking sequence data in the GenBank. While morphologies of *Tubeufia* species are quite similar, using morphology alone presents difficulties for identification; thus, sequence data are required to resolve taxonomic confusions. The last treatment of *Tubeufia* was provided by Lu et al. [3], and they introduced seventeen new species and six new combinations in the genus, accepting fifty species in the genus based on both phylogenic analysis and morphological characters. In this paper, we introduced two novel species, one new record species, and a new isolate of known species in *Tubeufia*.

**Figure 7:**
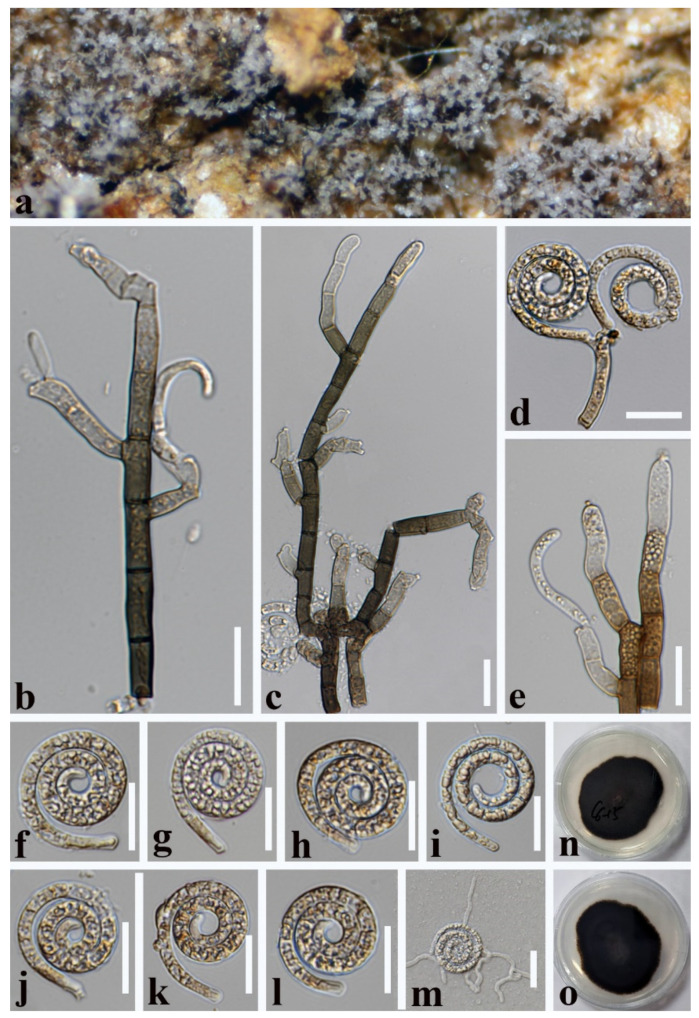
*Tubeufia cocois* (MFLU 21–0192, holotype). (**a**) Colony on decaying leaves of *Cocos nucifera*; (**b**,**c**,**e**) conidiophores and conidiogenous cells; (**d**) conidia and conidiogenous cells; (**f**–**l**) conidia; (**m**) germinated conidium; (**n**,**o**) colony cultures on PDA (observe and reverse). **Scale bars**: (**b**–**m**) = 20 µm.

***Tubeufia cocois*** X.G. Tian and Tibpromma, sp. nov. (Figure 7).

Index Fungorum number, IF 555070; Facesoffungi number, FoF 10576

Etymology: Referring to the host plant *Cocos nucifera*, on which the fungus was collected.

*Saprobic* on the decaying leaves of *Cocos nucifera*. **Sexual morph** Undetermined. **Asexual morph** Hyphomycetous, helicosporous. *Colonies* on the substratum are superficial, effuse, gregarious, and white to pale brown. *Mycelium* is partly immersed, partly superficial, hyaline to brown, septate, branched, and with glistening conidia. *Conidiophores* 38–123 × 4.5–6 µm ($\bar{x}$ = 80.5 × 5.5 µm, n = 20) are macronematous, mononematous, straight or slightly flexuous, cylindrical, branched, septate, orange brown to dark brown, paler towards the apex, and smooth-walled. *Conidiogenous cells* 8–17.5 × 4–5.5 µm ($\bar{x}$ = 13 × 5 µm, n =25) are holoblastic, mono- to polyblastic, integrated, sympodial, terminal or intercalary, irregular cylindrical, hyaline to pale brown, and smooth-walled, with most of them being denticulate protrusions. *Conidia* are solitary, acropleurogenous, helicoid, rounded at tip, with a 26–32.5 μm ($\bar{x}$ = 29 μm, n = 20) diam. and a conidial filament 3.5–5 μm ($\bar{x}$ = 4 μm, n = 20) wide, 116–136 μm ($\bar{x}$ = 126 μm, n = 20) long, and coiled 2¼–2¾ times, and they do not become loose in water, are indistinctly multi-septate, guttulate, hyaline when young, pale brown to brown at maturity, and smooth-walled.

Culture characteristics: conidia germinated on PDA within 12 h; colonies grow on PDA, reach 30 mm in 2 weeks at 28 °C, are irregular, with a flat surface, edge undulate, and brown to dark brown in PDA medium; mycelium are superficial and partially immersed, branched, septate, hyaline to brown, and smooth.

Material examined: Thailand, Chiang Rai Province, on decaying leaves of *Cocos nucifera*, 16 January 2021, X. G. Tian, C6–15 (MFLU 21–0192, holotype), ex-type culture, MFLUCC 22–0001; *ibid,* C6–8 (MFLU 21–0186, paratype), ex-paratype, MFLUCC 22–0002; *ibid,* C6–20 (MFLU 21–0187, paratype), ex-paratype, MFLUCC 22–0003.

Notes: *Tubeufia cocois* is introduced as a distinct new species from *Cocos nucifera* in Thailand. In the phylogenetic analyses, three newly obtained strains of *T. cocois* (MFLUCC 22–0001, MFLUCC 22–0002, and MFLUCC 22–0003) clustered together and were sister to three *T. aquatica* strains with strong statistical support values (100% ML/1.00 PP, Figure 1). Morphologically, *T. cocois* can be easily distinguished from *T. aquatica* by the shape and size of the conidiophores, conidiogenous cells, and conidia. *Tubeufia cocois* has branched or unbranched, multi-septate, and longer conidiophores (38–123 vs. 18–40 μm), while the conidiophores of *T. aquatica* are unbranched, 0–1-septate, and shorter than those of *T. cocois*. The conidiogenous cells of *T. cocois* are terminal or intercalary and conidia are acropleurogenous, whereases *T. aquatica* has terminal conidiogenous cells and conidia are acrogenous [53]. Based on pairwise nucleotide comparisons, the new strain (MFLUCC 22-0001) is different from *T. aquatica* (MFLUCC 16–1249) in 16/413 bp (3.87%) of the ITS, 2/845 (0.24%) of the LSU, 23/919 bp (2.5%) of RPB2, and 10/617 bp (1.62%) of TEF1-α.

**Figure 8:**
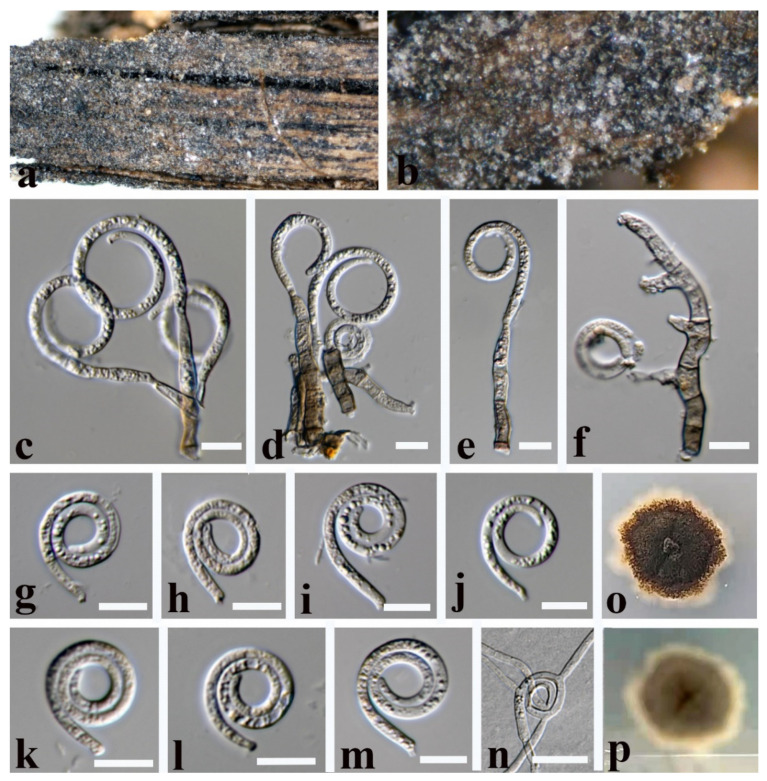
*Tubeufia laxispora* (MFLU 21–0191). (**a**,**b**) Colony on decaying leaves of *Cocos nucifera*; (**c**–**f**) conidiogenous cells and conidia; (**g**–**m**) conidia; (**n**) germinated conidium; (**o**,**p**) colony cultures on PDA (observe and reverse). **Scale bars**: (**c**–**n**) = 20 µm.

***Tubeufia laxispora*** Y.Z. Lu, Boonmee, and K.D. Hyde, Mycological Progress 16: 409 (2017) (Figure 8).

Index Fungorum, IF 818987; Facesoffungi number, FoF 02694

*Saprobic* on the decaying leaves of *Cocos nucifera*. **Sexual morph** Undetermined. **Asexual morph** Hyphomycetous, helicosporous. *Colonies* on the substratum are superficial, effuse, gregarious, and range from white to brown. *Mycelium* is partly immersed, partly superficial, pale brown, septate, sparsely branched hyphae, with masses of crowded conidia. *Conidiophores* 26–53 × 3.5–5 µm ($\bar{x}$ = 39.5 × 4 µm, n =15) are hyaline to brown, macronematous, erect, short, and smooth-walled. *Conidiogenous cells* 8–16 × 3–4.5 µm ($\bar{x}$ = 12 × 4 µm, n =20) are monoblastic, holoblastic, integrated, and each have a single conidium. *Conidia* are solitary, acropleurogenous, helicoid, and rounded at the tip, with a 17.5–29 μm ($\bar{x}$ = 23 μm, n = 20) diam. and a conidial filament 2–3.5 μm ($\bar{x}$ = 2.5 μm, n = 20) wide, 63–94.5 μm ($\bar{x}$ = 78.5 μm, n = 20) long, loosely coiled 1–2½ times in the water, indistinctly multi-septate, hyaline, and smooth-walled.

Culture characteristics: conidia germinating on PDA within 12 h; colonies growing on PDA, reaching 20 mm in 2 weeks at 28 °C—they are circular, with a flat surface, edge undulate, and brown to dark brown in PDA medium; mycelium are superficial and partially immersed, branched, septate, hyaline to brown, and smooth.

Material examined: Thailand, Chiang Rai Province, on decaying leaves of *Cocos nucifera*, 9 March 2021, X. G. Tian, C7–10 (MFLU 21–0191), living culture, MFLUCC 21–0163.

Notes: In our phylogenetic analyses, the newly obtained isolate (MFLUCC 21–0163) clustered with four strains of *T. laxispora* with high statistical supports (100% ML/1.00 PP, Figure 1). Based on pairwise nucleotide comparisons, the new strain (MFLUCC 21–0163) almost overlapped with the ex-type strain of *T. laxispora* (MFLUCC 16–0232), except TEF1-α 1 bp out of 878 bp (<1%). Morphologically, our new isolate fits well with the description of *T. laxispora,* except for the conidial size (17.5–40 μm diam., 63–94.5 μm long vs. 17.5–29 μm diam., 111–182 μm long) [37]. Hence, we identify our new isolate as *T. laxispora*. *Tubeufia laxispora* was described by Lu et al. [37] on submerged wood in Thailand, while our new isolate was collected on decaying leaves of *Cocos nucifera* in Thailand, and this is the first report of *Tubeufia laxispora* associated with a coconut tree from Thailand.

**Figure 9:**
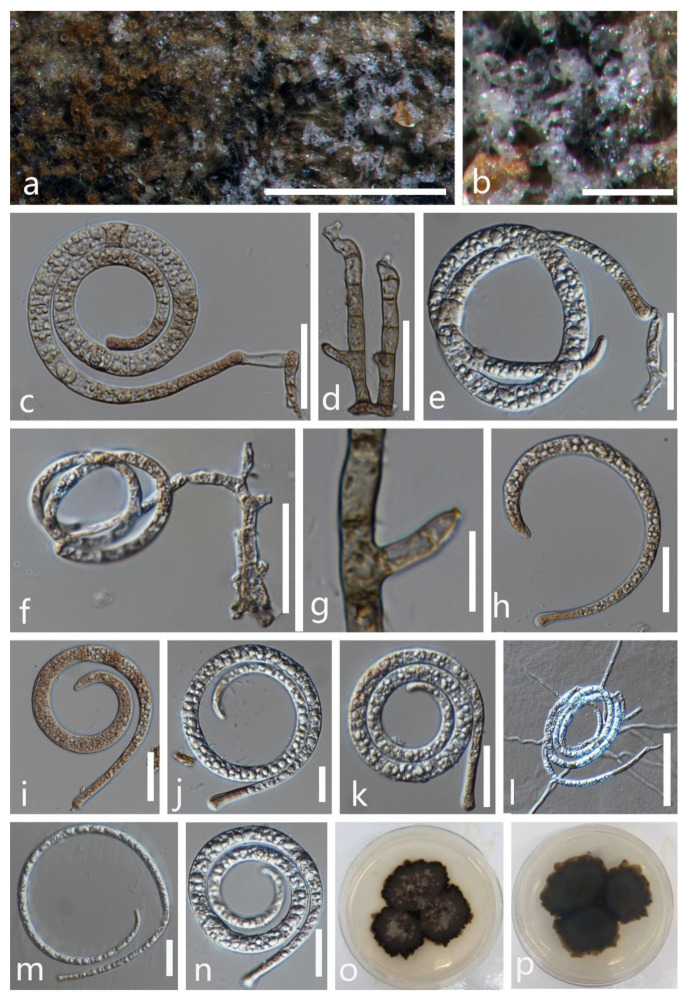
*Tubeufia cylindrothecia* (MFLU 21–0190). (**a**,**b**) Colony on decaying wood; (**c**,**e**,**f**) conidiophores with attached conidia; (**d**,**g**) conidiophores and conidiogenous cells; (**h**–**k**,**m**,**n**) conidia; (**l**) germinated conidium; (**o**,**p**) colony cultures on PDA (observe and reverse)**. Scale bars**: (**a**) = 1000 µm, (**b**) = 200 µm, (**l**) = 50 µm, (**c**–**f**,**h**) = 30 µm, (**i**–**k**,**m**,**n**) = 20 µm, (**g**) = 10 µm.

***Tubeufia cylindrothecia*** (Seaver) Höhn Sber. Akad. Wiss. Wien, Math. -naturw. Kl., Abt. 1 128: 562 (1919) (Figure 9).

Index Fungorum, IF 340543; Facesoffungi number, FoF 02650

Saprobic on submerged decaying wood. **Sexual morph** See Seaver [54]. **Asexual morph** *Colonies* on the substratum are superficial, effuse, gregarious, and white to pale brown. *Mycelium* is partly immersed, partly superficial, hyaline to brown, septate, and with masses of conidia. *Conidiophores* 57–95 × 4–7 µm ($\bar{x}$ = 76 × 5.5 µm, n =10) are pale brown, macronematous, mononematous, septate, cylindrical, unbranched, erect, and smooth-walled. *Conidiogenous cells* 6.5–16 × 3.5–5 µm ($\bar{x}$ = 11 × 4 µm, n =15) are holoblastic, monoblastic, integrated, smooth, terminal or intercalary, and cylindrical. *Conidia* are acropleurogenous, 40.5–82 μm ($\bar{x}$ = 61.5 μm, n = 20) in diam. and with a conidial filament that is 3.5–8 μm ($\bar{x}$ = 5.5 μm, n = 20) wide, 220–321 μm ($\bar{x}$ = 270.5 μm, n = 20) long, helicoid, with conidial loosely coiled 1–3½ times in the water, hyaline to brown, indistinctly multi-septate, guttulate, and smooth.

Culture characteristics: conidia germinating on PDA within 12 h; colonies growing on PDA, reaching 25 mm in 2 weeks at 28 °C, irregular, with a flat surface, edge undulate, and brown to dark brown in PDA medium; mycelium are partially immersed, branched, septate, hyaline to brown, and smooth.

Material examined: Thailand, Chiang Rai Province, on decaying submerged wood, 11 November 2020, X. G. Tian, W1–10 (MFLU 21–0190), living culture, MFLUCC 21–0160.

Notes: *Tubeufia cylindrothecia* was originally introduced with both sexual and asexual morphs that link to *Helicomyces roseus* based on morphological studies [6,55], while phylogenetic analyses showed that *Tubeufia cylindrotheci* and *Helicomyces roseus* can be recognized as two different species [1,8,53,56]. Luo et al. [53] first reported its asexual morph as collected from a freshwater habitat in China. Our phylogenetic results show that the newly obtained isolate (MFLUCC 21–0160) clustered with six strains of *T. cylindrothecia* with high bootstrap support (98% ML/1.00 PP). Morphologically, our new isolate is almost identical to *T. cylindrothecia,* except for the conidiogenous cells of the new isolate (MFLUCC 21–0160) that are terminal or intercalary, while the conidiogenous cells are terminal in *T. cylindrothecia* (MFLU 16–2547). Thus, based on morphological and molecular data, we identify the new isolate as *Tubeufia cylindrothecia*.

**Figure 10:**
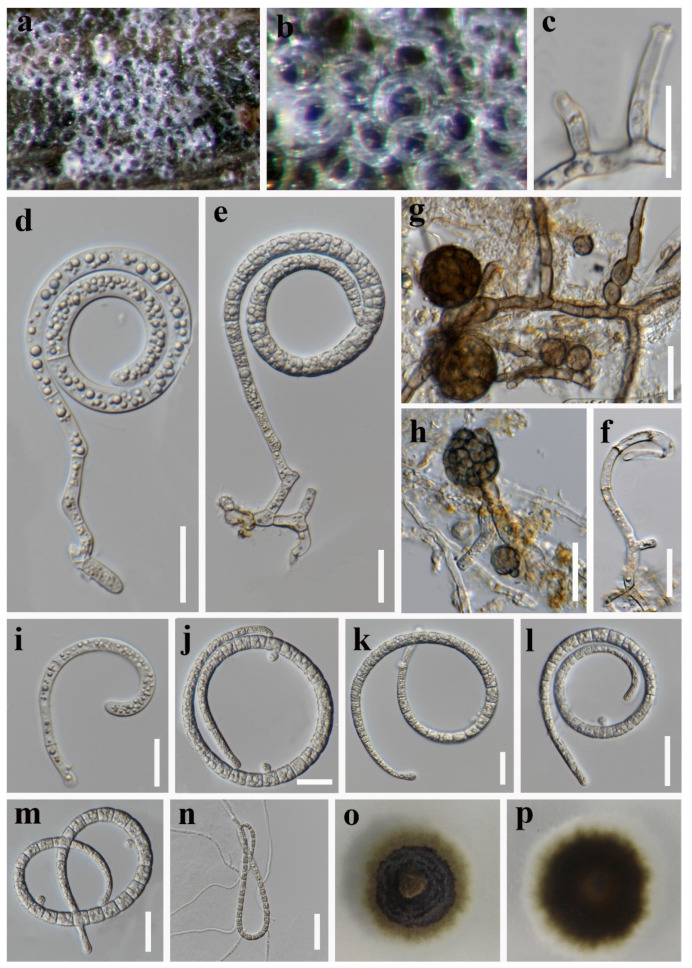
*Tubeufia longihelicospora* (MFLU 21–0182). (**a**,**b**) Colony on decaying wood; (**c**–**f**) conidiophores, conidiogenous cells, and conidia; (**g**,**h**) stalked sclerotia; (**i**–**m**) conidia; (**n**) germinated conidium; (**o**,**p**) colony cultures on PDA (observe and reverse). **Scale bars**: (**n**) = 40 µm, (**c**–**m**) = 20 µm.

***Tubeufia longihelicospora*** Boonmee, Promputtha, and K.D. Hyde, in Boonmee et al., Fungal Diversity 111: 133 (2021) (Figure 10).

Index Fungorum number, IF 558543; Facesoffungi number, FoF 09195

*Saprobic* on submerged decaying wood in a freshwater stream. **Sexual morph** Undetermined. **Asexual morph** Hyphomycetous, helicosporous. *Colonies* on the substratum are superficial, effuse, gregarious, and white. *Mycelium* is composed of partly immersed, hyaline to pale brown, septate, branched hyphae with masses of glistening, crowded conidia. Stalked sclerotia are often present and are medium brown, spherical, and muriform. *Conidiophores* (10.5–)13.5–35.5(–55.5) × 3.5–5(–6) μm ($\bar{x}$ = 24.5 × 4 μm, n = 15) are macronematous, mononematous, arise as lateral branches from creeping hyphae, cylindrical, branched, 0–4-septate, hyaline to pale brown, and smooth-walled. *Conidiogenous cells* (7–)9–19(–24.5) × 3.5–4.5 μm ($\bar{x}$ = 14 × 4 μm, n = 15) are holoblastic, monoblastic, integrated, terminal or intercalary, cylindrical, truncate at the apex after conidial secession, hyaline to pale brown, and smooth-walled. *Conidia* are solitary, acrogenous, holoblastic, helicoid, rounded at the tip, hyaline to pale brown, (40–)55.5–86.5(–100.5) μm ($\bar{x}$ = 71 μm, n = 15) in diam. and with a conidial filament 6.5–8.5 μm ($\bar{x}$ = 7.5 μm, n = 15) wide in the broadest part and tapering towards the ends, (99.5–)240.5–355.5 μm ($\bar{x}$ = 298 μm, n = 15) long, loosely coiled 1–2 times, multi-septate, tightly to loosely coiled in the water, constricted at the septa, guttulate, hyaline to pale brown, rough-walled, and bearing conidiola. Conidiola are globose, unicellular, and rough-walled.

Culture characteristics: conidia germinated on PDA within 12 h; colonies growing on PDA reach 20 mm in 2 weeks at 28 °C, are irregular, with a flat surface, edge undulate, and brown to dark brown in PDA medium; mycelium are superficial and partially immersed, branched, septate, hyaline to brown, and smooth.

Material examined: Thailand, Chiang Rai Province, Mueang, Ban Du, on decaying submerged wood, 15 August 2020, R. J. Xu, MD77 (MFLU 21–0182), living culture, MFLUCC 21–0151; China, Yunnan Province, Xishuangbanna, on decaying submerged wood, 13 September 2021, X. G. Tian, WB12 (HKAS 122173), living culture, KUMCC 21–0814; *ibid* NWBB9 (HKAS 122169), living culture, KUMCC 21–0815.

Notes: In the phylogenetic analyses, our three new strains (MFLUCC 21–0151, KUMCC 21–0478, and KUMCC 21–0479) are clustered together within the same clade as *T. longihelicospora* (MFLUCC 16–0753). Based on pairwise nucleotide comparisons, our three new strains almost overlap with the ex-type strain of *Tubeufia longihelicospora* (MFLUCC 16–0753). Morphologically, our new isolate (MFLU 21–0182) is almost identical to *Tubeufia longihelicospora,* except for the size of the conidia (55.5–86.5 vs. 36–52 µm diam.). Therefore, we identify the three new isolates as *Tubeufia longihelicospora* based on morphological and phylogenetic data. *Tubeufia longihelicospora* was introduced by Boonmee et al. [57] on a submerged decaying wood in a small freshwater stream in Thailand. Our isolate *Tubeufia longihelicospora* (MFLUCC 21–0151) was also collected in Thailand, while the other two isolates of *Tubeufia longihelicospora* (KUMCC 21–0478 and KUMCC 21–0479) were collected in China, which is a new geographical record.

## 4. Discussion

Tubeufiaceae is an interesting family with diverse morphologies, habitats, and a worldwide distribution [1,3,12,24]. The asexual morph of the Tubeufiaceae species is reported as helicosporous, chlamydosporous, and phragmosporous conidia, of which helicosporous conidia are the most common morphology in Tubeufiaceae. However, species with helicosporous conidia are not only found in Tubeufiaceae; for example, *Helicoascotaiwania* also has helicosporous conidia but it is phylogenetically distinct from Tubeufiaceae. *Helicoascotaiwania* is placed in Pleurotheciaceae and Sordariomycetes [58], while Tubeufiaceae is placed in Pleosporales. The interesting finding is that the asexual morphs of some genera were reported with two different morphologies. For example, *Tubeufia* and *Berkleasmium* produce both dictyosporous and helicosporous conidia, while *Helicoma* produces both helicosporous and phragmosporous conidia, confirmed by phylogenetic analyses [3].

The morphology of helicosporous hyphomycetes is quite similar; thus, the phylogenetic analyses are efficient to identify the helicosporous hyphomycetes at the species level [59,60,61]. With the availability of molecular data, some species were revised and transferred to other genera based on phylogenetic analyses. For example, *Helicomyces roseus* (BCC 3381) and *Helicoma perelegans* (ATCC 22621) were transferred to *Tubeufia* [3]. However, many helicosporous species lack sequence data in the GenBank, and the taxonomy of helicosporous hyphomycetes needs revisions based on phylogenetic analyses. For example, species in the genera of *Tubeufia*, *Helicoma,* and *Helicomyces* were introduced based on morphological characteristics, but many of them lack sequence data in the GenBank; thus, further study into herbarium specimens is necessary to resolve taxonomic problems in the three genera.

In this study, nine Tubeufiaceae species were collected from terrestrial and freshwater habitats in Thailand, of which three were introduced as new species, while six were identified as existing species based on phylogenetic analyses and morphological characteristics. The nine species were placed in *Dematiohelicomyces*, *Helicoma, Helicotruncatum*, *Neohelicosporium*, *Parahelicomyces*, and *Tubeufia*, respectively, of which the genera *Dematiohelicomyces*, *Helicotruncatum*, *Neohelicosporium,* and *Parahelicomyces* are well studied, and all species in these genera have sequence data available in the GenBank. *Helicoma* and *Tubeufia* were recently revised by Lu et al. [3]. In our phylogenetic analyses, *Helicoma* and *Tubeufia* formed well-supported and monophyletics clades within the family. The morphologies of *Tubeufia* and *Helicoma* are quite similar; thus, morphology alone is not enough to identify species in *Tubeufia* and *Helicoma*, and phylogenetic analyses are necessary. However, earlier studies identified the two genera only based on morphological characteristics, and sequence data of many species are not available in the GenBank, so, it is entirely possible that some species were incorrectly identified; accordingly, fresh collections and molecular data are required to clarify their taxonomic status. Even though *Dematiohelicomyces helicosporus*, *Helicotruncatum palmigenum*, *Helicoma guttulatum*, *Neohelicosporium bambusicola*, *Tubeufia cylindrothecia, T. laxispora*, and *T. longihelicospora* are known species and were collected again, some species are known as new hosts and new geographical records. In addition, it is also better to provide the full descriptions and color plates of the micro-characteristics of new isolates to understand some fine morphological differences.

## Figures and Tables

**Table 1:** Names, culture collection accession numbers, and corresponding GenBank accession numbers of the fungal taxa used in this study.

Taxa	Strain Numbers	GenBank Accession Numbers			
		ITS	LSU	TEF1-α	RPB2
*Aquaphila albicans*	BCC 3543	DQ341096	DQ341101	–	–
*Aquaphila albicans*	MFLUCC 16–0010	KX454165	KX454166	KY117034	MF535255
*Berkleasmium concinnum*	ILLS 80803	KY582485	–	–	–
*Berkleasmium fusiforme*	MFLUCC 17–1979	MH558694	MH558821	MH550885	MH551008
*Berkleasmium longisporum*	MFLUCC 17–1990	MH558697	MH558824	MH550888	MH551011
*Botryosphaeria agaves* ^T^	MFLUCC 10–0051	JX646790	JX646807	JX646855	–
*Botryosphaeria dothidea*	CBS 115476	–	NG_027577	–	–
*Chlamydotubeufia cylindrica* ^T^	MFLUCC 16–1130	MH558702	MH558830	MH550893	MH551018
*Chlamydotubeufia huaikangplaensis* ^T^	MFLUCC10–0926	JN865210	JN865198	–	–
*Chlamydotubeufia krabiensis* ^T^	MFLUCC 16–1134	KY678767	KY678759	KY792598	MF535261
*Dematiohelicomyces helicosporus* ^T^	MFLUCC 16–0213	KX454169	KX454170	KY117035	MF535258
*Dematiohelicomyces helicosporus*	MFLUCC 16–0003	MH558703	MH558831	MH550894	MH551019
*Dematiohelicomyces helicosporus*	MFLUCC 16–0007	MH558704	MH558832	MH550895	MH551020
***Dematiohelicomyces helicosporus***	**KUMCC 21–0473**	**OM331856**	**OL985958**	**OM355487**	–
*Dictyospora thailandica*	MFLUCC 16–0215	KY873628	KY873623	KY873287	–
*Dictyospora thailandica*	MFLUCC 11–0512	KF301528	KF301536	–	–
*Helicoarctatus aquaticus* ^T^	MFLUCC 17–1996	MH558707	MH558835	MH550898	MH551024
*Helicodochium aquaticum*	MFLUCC 16–0008	MH558708	MH558836	MH550899	MH551025
*Helicodochium aquaticum* ^T^	MFLUCC 17–2016	MH558709	MH558837	MH550900	MH551026
*Helicohyalinum aquaticum* ^T^	MFLUCC 16–1131	KY873625	KY873620	KY873284	MF535257
*Helicohyalinum infundibulum* ^T^	MFLUCC 16–1133	MH558712	MH558840	MH550903	MH551029
*Helicoma ambiens*	UAMH 10533	AY916451	AY856916	–	–
*Helicoma ambiens*	UAMH 10534	AY916450	AY856869	–	–
*Helicoma aquaticum* ^T^	MFLUCC 17–2025	MH558713	MH558841	MH550904	MH551030
*Helicoma brunneisporum* ^T^	MFLUCC 17–1983	MH558714	MH558842	MH550905	MH551031
*Helicoma dennisii*	NBRC 30667	AY916455	AY856897	–	–
*Helicoma fusiforme* ^T^	MFLUCC 17–1981	MH558715	–	MH550906	–
*Helicoma guttulatum* ^T^	MFLUCC 16–0022	KX454171	KX454172	MF535254	MH551032
***Helicoma guttulatum***	**MFLUCC 21** **–0152**	**OL545456**	**OL606150**	**OL964521**	**OL964527**
*Helicoma hongkongense*	MFLUCC 17–2005	MH558716	MH558843	MH550907	MH551033
*Helicoma inthanonense* ^T^	MFLUCC 11–0003	JN865211	JN865199	–	–
*Helicoma khunkornensis* ^T^	MFLUCC 10–0119	JN865203	JN865191	KF301559	_
*Helicoma linderi*	NBRC 9207	AY916454	AY856895	–	–
*Helicoma longisporum*	MFLUCC 16–0002	MH558717	MH558844	MH550908	MH551034
*Helicoma longisporum*	MFLUCC 16–0005	MH558718	–	MH550909	MH551035
*Helicoma longisporum*	MFLUCC 16–0211	MH558719	MH558845	MH550910	MH551036
*Helicoma longisporum* ^T^	MFLUCC 17–1997	MH558720	MH558846	MH550911	MH551037
*Helicoma miscanthi* ^T^	MFLUCC 11–0375	KF301525	KF301533	KF301554	–
*Helicoma muelleri*	CBS 964.69	AY916453	AY856877	–	–
*Helicoma muelleri*	UBC F13877	AY916452	AY856917	–	–
*Helicoma multiseptatum* ^T^	GZCC 16–0080	MH558721	MH558847	MH550912	MH551038
*Helicoma nematosporum* ^T^	MFLUCC 16–0011	MH558722	MH558848	MH550913	MH551039
*Helicoma rubriappendiculatum* ^T^	MFLUCC 18–0491	MH558723	MH558849	MH550914	MH551040
*Helicoma rufum* ^T^	MFLUCC 17–1806	MH558724	MH558850	MH550915	–
*Helicoma rugosum*	ANM 196	GQ856138	GQ850482	–	–
*Helicoma rugosum*	ANM 953	GQ856139	GQ850483	–	–
*Helicoma rugosum*	ANM 1169	–	GQ850484	–	–
*Helicoma rugosum*	JCM 2739	–	AY856888	–	–
*Helicoma septoconstrictum*	MFLUCC 17–1991	MH558725	MH558851	MH550916	MH551041
*Helicoma septoconstrictum* ^T^	MFLUCC 17–2001	MH558726	MH558852	MH550917	MH551042
*Helicoma siamense* ^T^	MFLUCC 10–0120	JN865204	JN865192	KF301558	_
*Helicoma* sp.	HKUCC 9118	–	AY849966	–	–
*Helicoma tectonae* ^T^	MFLUCC 12–0563	KU144928	KU764713	KU872751	_
*Helicoma vaccinii*	CBS 216.90	AY916486	AY856879	_	_
*Helicomyces chiayiensis* ^T^	BCRC FU30842	LC316604	–	–	–
*Helicomyces colligatus*	MFLUCC 16–1132	MH558727	MH558853	MH550918	MH551043
*Helicomyces hyalosporus*	GZCC 16–0070	MH558728	MH558854	MH550919	MH551044
*Helicomyces hyalosporus*	MFLUCC 17–0051	MH558731	MH558857	MH550922	MH551047
*Helicomyces torquatus*	MFLUCC 16–0217	MH558732	MH558858	MH550923	MH551048
*Helicosporium flavum* ^T^	MFLUCC 16–1230	KY873626	KY873621	KY873285	–
*Helicosporium luteosporum* ^T^	MFLUCC 16–0226	KY321324	KY321327	KY792601	MH551056
*Helicosporium vesicarium* ^T^	MFLUCC 17–1795	MH558739	MH558864	MH550930	MH551055
*Helicotruncatum palmigenum*	NBRC 32663	AY916480	AY856898	–	–
*Helicotruncatum palmigenum*	MFLUCC 15–0993	MT627685	MN913690	–	–
***Helicotruncatum palmigenum***	**KUMCC 21–0474**	**OM102542**	**OL985959**	**OM355488**	**OM355492**
*Helicotubeufia guangxiensis* ^T^	MFLUCC 17–0040	MH290018	MH290023	MH290028	MH290033
*Helicotubeufia hydei* ^T^	MFLUCC 17–1980	MH290021	MH290026	MH290031	MH290036
*Helicotubeufia jonesii* ^T^	MFLUCC 17–0043	MH290020	MH290025	MH290030	MH290035
*Muripulchra aquatica*	DLUCC 0571	KY320531	KY320548	–	–
*Muripulchra aquatica*	KUMCC 15–0245	KY320533	KY320550	KY320563	MH551057
*Muripulchra aquatica*	KUMCC 15–0276	KY320534	KY320551	KY320564	MH551058
*Muripulchra aquatica* ^T^	MFLUCC 15–0249	KY320532	KY320549	–	–
*Neoacanthostigma fusiforme* ^T^	MFLUCC 11–0510	KF301529	KF301537	–	–
*Neochlamydotubeufia fusiformis* ^T^	MFLUCC 16–0016	MH558740	MH558865	MH550931	MH551059
*Neochlamydotubeufia fusiformis*	MFLUCC 16–0214	MH558741	MH558866	MH550932	MH551060
*Neochlamydotubeufia khunkornensis* ^T^	MFLUCC 10–0118	JN865202	JN865190	KF301564	–
*Neochlamydotubeufia khunkornensis*	MFLUCC 16–0025	MH558742	MH558867	MH550933	MH551061
*Neohelicomyces aquaticus*	KUMCC 15–0463	KY320529	KY320546	KY320562	MH551065
*Neohelicomyces grandisporus* ^T^	KUMCC 15–0470	KX454173	KX454174	–	MH551067
*Neohelicomyces submersus* ^T^	MFLUCC 16–1106	KY320530	KY320547	–	MH551068
*Neohelicosporium abuense*	CBS 101688	AY916470	–	–	–
*Neohelicosporium acrogenisporum* ^T^	MFLUCC 17–2019	MH558746	MH558871	MH550937	MH551069
*Neohelicosporium aquaticum* ^T^	MFLUCC 17–1519	MF467916	MF467929	MF535242	MF535272
*Neohelicosporium astrictum* ^T^	MFLUCC 17–2004	MH558747	MH558872	MH550938	MH551070
*Neohelicosporium aurantiellum*	ANM 718	GQ856140	GQ850485	–	–
***Neohelicosporium bambusicola* ^T^**	**MFLUCC 21–0156**	**OL606157**	**OL606146**	**OL964517**	**OL964523**
*Neohelicosporium ellipsoideum* ^T^	MFLUCC 16–0229	MH558748	MH558873	MH550939	MH551071
*Neohelicosporium fusisporum* ^T^	MFUCC 16–0642	MG017612	MG017613	MG017614	–
*Neohelicosporium griseum*	CBS 961.69	AY916474	AY856884	–	–
*Neohelicosporium griseum*	CBS 113542	AY916475	AY916088	–	–
*Neohelicosporium guangxiense*	GZCC 16–0042	MF467920	MF467933	MF535246	MF535276
*Neohelicosporium guangxiense*	MFLUCC 17–0054	MH558750	MH558875	MH550941	MH551073
*Neohelicosporium hyalosporum*	GZCC 16–0063	MH558751	MH558876	MH550942	MH551074
*Neohelicosporium hyalosporum* ^T^	GZCC 16–0076	MF467923	MF467936	MF535249	MF535279
*Neohelicosporium irregulare* ^T^	MFLUCC 17–1796	MH558752	MH558877	MH550943	MH551075
*Neohelicosporium irregulare*	MFLUCC 17–1808	MH558753	MH558878	MH550944	MH551076
*Neohelicosporium krabiense* ^T^	MFLUCC 16–0224	MH558754	MH558879	MH550945	MH551077
*Neohelicosporium laxisporum* ^T^	MFLUCC 17–2027	MH558755	MH558880	MH550946	MH551078
*Neohelicosporium morganii*	CBS 281.54	AY916468	AY856876	–	–
*Neohelicosporium morganii*	CBS 222.58	AY916469	AY856880	–	–
*Neohelicosporium ovoideum* ^T^	GZCC 16–0064	MH558756	MH558881	MH550947	MH551079
*Neohelicosporium ovoideum*	GZCC 16–0066	MH558757	MH558882	MH550948	MH551080
*Neohelicosporium panacheum*	CBS 257.59	AY916471	AY916087	–	–
*Neohelicosporium parvisporum*	GZCC 16–0078	MF467924	MF467937	MF535250	MF535280
*Neohelicosporium parvisporum*	MFLUCC 17–2010	MH558763	MH558888	MH550954	MH551086
*Neohelicosporium* sp.	CBS 189.95	AY916472	AY856882	–	–
*Neohelicosporium* sp.	HKUCC 10235	–	AY849942	–	–
*Neohelicosporium submersum*	MFLUCC 17–2376	MT627738	MN913738	–	–
*Neohelicosporium taiwanense* ^T^	BCRC FU30841	LC316603	–	–	–
*Neohelicosporium thailandicum* ^T^	MFLUCC 16–0221	MF467928	MF467941	MF535253	MF535283
*Neotubeufia krabiensis* ^T^	MFLUCC 16–1125	MG012031	MG012024	MG012010	MG012017
*Parahelicomyces aquaticus* ^T^	MFLUCC 16–0234	MH558766	MH558891	MH550958	MH551092
***Parahelicomyces chiangmaiensis* ^T^**	**MFLUCC 21** **–0159**	**OL697884**	**OL606145**	**OL964516**	**OL964522**
*Parahelicomyces hyalosporus*	CBS 283.51	AY916464	AY856881	DQ677928	DQ677981
*Parahelicomyces hyalosporus*	KUMCC 15–0411	KY320527	KY320544	KY320560	–
*Parahelicomyces hyalosporus* ^T^	MFLUCC 15–0343	KY320523	KY320540	–	–
*Parahelicomyces indicus*	CBS 374.93	AY916477	AY856885	–	–
*Parahelicomyces menglunicus* ^T^	HKAS 85793	MK335914	–	MK335916	
*Parahelicomyces paludosus*	CBS 120503	DQ341095	DQ341103	–	–
*Parahelicomyces quercus*	MFLU 18-2091	–	–	MK360077	MK434906
*Parahelicomyces quercus*	MFUCC 17-0895	MK347720	MK347934	–	–
*Parahelicomyces talbotii*	MUCL 33010	AY916465	AY856874	–	–
*Parahelicomyces talbotii*	MFLUCC 17–2021	MH558765	MH558890	MH550957	MH551091
*Pseudohelicoon gigantisporum*	BCC 3550	AY916467	AY856904	–	–
*Pseudohelicoon subglobosum* ^T^	BCRC FU30843	LC316607	LC316610	–	–
*Thaxteriellopsis lignicola*	MFLUCC 10–0123	JN865207	JN865195	KF301562	_
*Thaxteriellopsis lignicola*	MFLUCC 10–0124	JN865208	JN865196	KF301561	_
*Tubeufia abundata* ^T^	MFLUCC 17–2024	MH558769	MH558894	MH550961	MH551095
*Tubeufia aquatica*	MFLUCC 17–1794	MH558770	MH558895	MH550962	MH551096
*Tubeufia aquatica* ^T^	MFLUCC 16–1249	KY320522	KY320539	KY320556	MH551142
*Tubeufia aquatica*	DLUCC 0574	KY320521	KY320538	KY320555	MH551141
*Tubeufia bambusicola* ^T^	MFLUCC 17–1803	MH558771	MH558896	MH550963	MH551097
*Tubeufia brevis* ^T^	MFLUCC 17–1799	MH558772	MH558897	MH550964	MH551098
*Tubeufia brunnea* ^T^	MFLUCC 17–2022	MH558773	MH558898	MH550965	MH551099
*Tubeufia chiangmaiensis*	MFLUCC 17–1801	MH558774	MH558899	MH550966	MH551100
*Tubeufia chiangmaiensis* ^T^	MFLUCC 11–0514	KF301530	KF301538	KF301557	–
*Tubeufia chlamydospora* ^T^	MFLUCC 16–0223	MH558775	MH558900	MH550967	MH551101
***Tubeufia cocois* ^T^**	**MFLUCC 22–0001**	**OM102541**	**OL985957**	**OM355486**	**OM355491**
***Tubeufia cocois***	**MFLUCC 22–0002**	**OM102543**	**OL985960**	**OM355489**	**OM355493**
***Tubeufia cocois***	**MFLUCC 22–0003**	**OM102544**	**OL985961**	**OM355490**	**OM355494**
*Tubeufia cylindrothecia*	BCC 3559	–	AY849965	–	–
*Tubeufia cylindrothecia*	BCC 3585	AY916482	AY856908	–	–
*Tubeufia cylindrothecia*	DLUCC 0572	KY320520	KY320537	KY320554	–
*Tubeufia cylindrothecia*	MFLUCC 16–1253	KY320519	KY320536	KY320553	–
*Tubeufia cylindrothecia*	MFLUCC 16–1283	KY320518	KY320535	KY320552	MH551143
***Tubeufia cylindrothecia***	**MFLUCC 21–0160**	**OL545365**	**OL606147**	**OL964518**	**OL964524**
*Tubeufia cylindrothecia*	MFLUCC 17–1792	MH558776	MH558901	MH550968	MH551102
*Tubeufia dictyospora* ^T^	MFLUCC 17–1805	MH558778	MH558903	MH550970	–
*Tubeufia dictyospora*	MFLUCC 16–0220	MH558777	MH558902	MH550969	MH551103
*Tubeufia eccentrica* ^T^	MFLUCC 17–1524	MH558782	MH558907	MH550974	MH551108
*Tubeufia eccentrica*	GZCC 16–0035	MH558779	MH558904	MH550971	MH551105
*Tubeufia entadae*	MFLU 18–2102	NR_163323	–	–	–
*Tubeufia entadae*	MFLU 18-2102	MK347727	MK347943	–	–
*Tubeufia fangchengensis* ^T^	MFLUCC 17–0047	MH558783	MH558908	MH550975	MH551109
*Tubeufia filiformis* ^T^	MFLUCC 16–1128	–	KY092407	KY117028	MF535284
*Tubeufia filiformis*	MFLUCC 16–1135	KY092416	KY092411	KY117032	MF535285
*Tubeufia filiformis*	MFLUCC 16–0236	–	MH558938	MH550976	MH551110
*Tubeufia geniculata* ^T^	BCRC FU30849	LC335817	–	–	–
*Tubeufia geniculata*	NCYU U2–1B	LC335816	–	–	–
*Tubeufia guangxiensis*	MFLUCC 17–0046	MH558784	MH558909	MH550977	MH551111
*Tubeufia hechiensis* ^T^	MFLUCC 17–0052	MH558785	MH558910	MH550978	MH551112
*Tubeufia hyalospora* ^T^	MFLUCC 15–1250	MH558786	MH558911	MH550979	–
*Tubeufia inaequalis*	GZCC 16–0079	MH558787	MH558912	MH550980	MH551113
*Tubeufia inaequalis*	MFLUCC 17–1998	MH558791	MH558916	MH550984	MH551117
*Tubeufia inaequalis*	BCC 8808	AY916481	AY856910	–	–
*Tubeufia javanica*	MFLUCC 12–0545	KJ880034	KJ880036	KJ880037	–
*Tubeufia krabiensis* ^T^	MFLUCC 16–0228	MH558792	MH558917	MH550985	MH551118
*Tubeufia latispora* ^T^	MFLUCC 16–0027	KY092417	KY092412	KY117033	MH551119
*Tubeufia laxispora*	MFLUCC 16–0013	MH558793	MH558918	MH550986	MH551120
*Tubeufia laxispora*	MFLUCC 16–0219	KY092414	KY092409	KY117030	MF535286
*Tubeufia laxispora* ^T^	MFLUCC 16–0232	KY092413	KY092408	KY117029	MF535287
*Tubeufia laxispora*	MFLUCC 17–2023	MH558794	MH558919	MH550987	MH551121
***Tubeufia laxispora***	**MFLUCC 21–0163**	**OL545455**	**OL606148**	**OL964519**	**OL964525**
*Tubeufia lilliputea*	NBRC 32664	AY916483	AY856899	–	–
*Tubeufia longihelicospora* ^T^	MFLUCC 16–0753	MZ538531	MZ538565	MZ567106	–
***Tubeufia longihelicospora***	**MFLUCC 21–0151**	**OL606156**	**OL606149**	**OL964520**	**OL964526**
***Tubeufia longihelicospora***	**KUMCC 21–0814**	**OM331690**	**OM331688**	**OM355484**	–
***Tubeufia longihelicospora***	**KUMCC 21–0815**	**OM331691**	**OM331705**	**OM355485**	–
*Tubeufia longiseta* ^T^	MFLUCC 15–0188	KU940133	–	–	–
*Tubeufia mackenziei* ^T^	MFLUCC 16–0222	KY092415	KY092410	KY117031	MF535288
*Tubeufia parvispora*	MFLUCC 17–1992	MH558796	MH558921	MH550989	MH551123
*Tubeufia parvispora*	MFLUCC 17–2003	MH558797	MH558922	MH550990	MH551124
*Tubeufia parvispora*	MFLUCC 17–2009	MH558798	MH558923	MH550991	MH551125
*Tubeufia roseohelicospora*	MFLUCC 16–0230	MH558799	MH558924	MH550992	MH551126
*Tubeufia roseohelicospora*	MFLUCC 17–1797	MH558800	MH558925	MH550993	MH551127
*Tubeufia roseohelicospora* ^T^	MFLUCC 15–1247	KX454177	KX454178	–	MH551144
*Tubeufia rubra*	GZCC 16–0083	MH558802	MH558927	MH550995	MH551129
*Tubeufia rubra* ^T^	GZCC 16–0081	MH558801	MH558926	MH550994	MH551128
*Tubeufia sahyadriensis* ^T^	NFCCI 4252	MH033849	MH033850	MH033851	–
*Tubeufia sessilis* ^T^	MFLUCC 16–0021	MH558803	–	MH550996	MH551130
*Tubeufia sympodihylospora* ^T^	MFLUCC 17–0044	MH558806	MH558930	MH550999	MH551133
*Tubeufia sympodilaxispora*	BCC 3580	–	DQ296554	–	–
*Tubeufia sympodilaxispora*	GZCC 16–0058	MH558807	MH558931	MH551000	MH551134
*Tubeufia sympodilaxispora* ^T^	MFLUCC 17–0048	MH558808	MH558932	MH551001	MH551135
*Tubeufia taiwanensis* ^T^	BCRC FU30844	LC316605	–	–	–
*Tubeufia tectonae*	MFLUCC 16–0235	MH558809	MH558933	MH551002	MH551136
*Tubeufia tectonae*	MFLUCC 17–1985	MH558810	MH558934	MH551003	MH551137
*Tubeufia tectonae* ^T^	MFLUCC 12–0392	KU144923	KU764706	KU872763	–
*Tubeufia tratensis* ^T^	MFLUCC 17–1993	MH558811	MH558935	MH551004	MH551138
*Tubeufia xylophila*	MFLUCC 17–1520	MH558813	MH558937	MH551006	MH551140
*Tubeufia xylophila*	GZCC 16–0038	MH558812	MH558936	MH551005	MH551139

Notes: Ex-type strains are indicated by **^T^** after the species name. Newly generated sequences are in black bold. The symbol “–” indicates information not available. Abbreviations: ANM, A.N. Miller; BCC, Biotec Culture Collection, Thailand; BCRC, Bioresearch Collection and Research Centre; CBS, Westerdijk Fungal Biodiversity Institute; DLUCC, Culture collection of Dali University; GUCC, Guizhou University Culture Collection; HKAS, the herbarium of Cryptogams Kunming Institute of Botany Academia Sinica; HKUCC, Hong Kong University Culture Collection; JCM, Japan Collection of Microorganisms; KUMCC, Culture collection of Kunming Institute of Botany; MFLU, the herbarium of the Mae Fah Luang University; MFLUCC, Mae Fah Luang University Culture Collection; NBRC, NITE Biological Resource Center; NCYU, National Chiayi University; NFCCI, National Fungal Culture Collection of India; UAMH, the University of Alberta Microfungus Collection and Herbarium; UBC F, University of British Columbia Herbarium.

## Data Availability

Not applicable.

## References

[1] Boonmee S., Rossman A.Y., Liu J.K., Li W.J., Dai D.Q., Bhat J.D., Jones E.B.G., McKenzie E.H.C., Xu J.C., Hyde K.D. (2014). Tubeufiales, ord. nov., integrating sexual and asexual generic names. Fungal Divers..

[2] Liu J.K., Hyde K.D., Jeewon R., Phillips A.J., Maharachchikumbura S.S., Ryberg M., Liu Z.Y., Zhao Q. (2017). Ranking higher taxa using divergence times: A case study in Dothideomycetes. Fungal Divers..

[3] Lu Y.Z., Liu J.K., Hyde K.D., Jeewon R., Kang J.C., Fan C., Boonmee S., Bhat D.J., Luo Z.L., Lin C.G. (2018). A taxonomic reassessment of Tubeufiales based on multi-locus phylogeny and morphology. Fungal Divers..

[4] Hongsanan S., Hyde K.D., Phookamsak R., Wanasinghe D.N., McKenzie E.H., Sarma V.V., Lücking R., Boonmee S., Bhat J.D., Liu N.G. (2020). Refined families of Dothideomycetes: Orders and families incertae sedis in Dothideomycetes. Fungal Divers..

[5] Barr M.E. (1979). A classification of Loculoascomycetes. Mycologia.

[6] Barr M.E. (1980). On the family Tubeufiaceae (Pleosporales). Mycotaxon.

[7] Rossman A.Y. (1987). The Tubeufiaceae and Similar Loculoascomycetes.

[8] Boonmee S., Zhang Y., Chomnunti P., Chukeatirote E., Tsui C.K., Bahkali A.H., Hyde K.D. (2011). Revision of lignicolous Tubeufiaceae based on morphological reexamination and phylogenetic analysis. Fungal Divers..

[9] Brahmanage R.S., Lu Y.Z., Bhat D.J., Wanasinghe D.N., Yan J.Y., Hyde K.D., Boonmee S. (2017). Phylogenetic investigations on freshwater fungi in Tubeufiaceae (Tubeufiales) reveals the new genus *Dictyospora* and new species *Chlamydotubeufia aquatica* and *Helicosporium flavum*. Mycosphere.

[10] Lu Y.Z., Boonmee S., Liu J.K., Hyde K.D., McKenzie E.H., Eungwanichayapant P.D., Kang J.C. (2018). Multi-gene phylogenetic analyses reveals *Neohelicosporium* gen. nov. and five new species of helicosporous hyphomycetes from aquatic habitats. Mycol. Prog..

[11] Rajeshkumar K.C., Sharma R. (2013). *Tamhinispora*, a new genus belongs to family Tubeufiaceae from the western Ghats, India based on morphology and phylogenetic analysis. Mycosphere.

[12] Dong W., Wang B., Hyde K.D., McKenzie E.H.C., Raja H.A., Tanaka K., Abdel-Wahab M.A., Abdel-Aziz F.A., Doilom M., Phookamsak R. (2020). Freshwater Dothideomycetes. Fungal Divers..

[13] Sivanesan A. (1984). The Bitunicate Ascomycetes and Their Anamorphs.

[14] Crane J.L., Shearer C.J., Barr M.E. (1998). A revision of *Boerlagiomyces* with notes and a key to the saprobic genera of Tubeufiaceae. Can. J. Bot..

[15] Kirk P.M., Cannon P.F., David J.C., Stalpers J.A. (2001). Ainsworth & Bisby’s Dictionary of the Fungi.

[16] Eriksson O.E., Winka K. (1998). Families and higher taxa of Ascomycota. Myconet.

[17] Eriksson O.E. (1999). Outline of Ascomycota—1999. Myconet.

[18] Eriksson O.E. (2001). SSU rDNA sequences from Ascomycota. Myconet.

[19] Eriksson O.E. (2005). Outline of Ascomycota—2005. Myconet.

[20] Lumbsch H.T., Huhndorf S.M. (2010). Myconet volume 14. Part One. Outline of Ascomycota—2009. Part Two. Notes on Ascomycete systematics. Nos. 4751–5113. Fieldiana Life Earth Sci..

[21] Kodsueb R., Jeewon R., Vijaykrishna D., McKenzie E.H.C., Lumyong P., Lumyong S., Hyde K.D. (2006). Systematic revision of Tubeufiaceae based on morphological and molecular data. Fungal Divers..

[22] Chaiwan N., Lu Y.Z., Tibpromma S., Bhat D.J., Hyde K.D., Boonmee S. (2017). *Neotubeufia* gen. nov. and *Tubeufia guangxiensis* sp. nov. (Tubeufiaceae) from freshwater habitats. Mycosphere.

[23] Hanada T., Sato T., Arioka M., Uramoto M., Yamasaki M. (1996). Purification and characterization of a 15 kDa protein (p15) produced by *Helicosporium* that exhibits distinct effects on neurite outgrowth from cortical neurons and PC12 cells. Biochem. Biophys. Res. Commun..

[24] Hu H., Guo H., Li E., Liu X., Zhou Y., Che Y. (2006). Decaspirones F–I, bioactive secondary metabolites from the saprophytic fungus *Helicoma viridis*. J. Nat. Prod..

[25] Itazaki H., Nagashima K., Sugita K., Yoshida H., Kawamura Y., Yasuda Y., Matsumoto K., Ishii K., Uotani N., Nakai H. (1990). Solation and structural elucidation of new cyclotetrapeptides, trapoxins A and B, having detransformation activities as antitumor agents. J. Antibiot..

[26] Tibpromma S., Hyde K.D., Jeewon R., Maharachchikumbura S.S., Liu J.K., Bhat D.J., Jones E.G., McKenzie E.H., Camporesi E., Bulgakov T.S. (2017). Fungal diversity notes 491–602: Taxonomic and phylogenetic contributions to fungal taxa. Fungal Divers..

[27] Tian X.G., Karunarathna S.C., Mapook A., Xu J.C., Bao D.F., Promputtha I., Tibpromma S. (2021). *Koorchaloma oryzae* sp. nov. (Stachybotryaceae, Sordariomycetes), from *Oryza sativa* (Poaceae) in northern Thailand. Phytotaxa.

[28] Tian X.G., Karunarathna S.C., Mapook A., Promputtha I., Xu J.C., Bao D.F., Tibpromma S. (2021). One new species and two new host records of *Apiospora* from bamboo and maize in northern Thailand with thirteen new combinations. Life.

[29] Senanayake I.C., Rathnayaka A.R., Marasinghe D.S., Calabon M.S., Gentekaki E., Lee H.B., Hurdeal V.G., Pem D., Dissanayake L.S., Wijesinghe S.N. (2020). Morphological approaches in studying fungi: Collection, examination, isolation, sporulation and preservation. Mycosphere.

[30] (2021). Index Fungorum. http://www.indexfungorum.org/Names/Names.asp.

[31] Jayasiri S.C., Hyde K.D., Ariyawansa H.A., Bhat J., Buyck B., Cai L., Dai Y.C., Abd-Elsalam K.A., Ertz D., Hidayat I. (2015). The faces of fungi database: Fungal names linked with morphology, phylogeny and human impacts. Fungal Divers..

[32] White T.J., Bruns T., Lee S., Taylor J., Innis G.M., Shinsky D., White T. (1990). Amplification and direct sequencing of fungal ribosomal RNA genes for phylogenetics. PCR Protocols: A Guide to Methods and Applications.

[33] Vilgalys R., Hester M. (1990). Rapid genetic identification and mapping of enzymatically amplified ribosomal DNA from several *Cryptococcus* species. J. Bacteriol..

[34] Rehner S.A., Buckley E. (2005). A Beauveria phylogeny inferred from nuclear ITS and EF1-α sequences: Evidence for cryptic diversification and links to *Cordyceps* teleomorphs. Mycologia.

[35] Liu Y.J., Whelen S., Hall B.D. (1999). Phylogenetic relationships among ascomycetes: Evidence from an RNA polymerse II subunit. Mol. Biol. Evol..

[36] Cai L., Jeewon R., Hyde K.D. (2005). Phylogenetic evaluation and taxonomic revision of *Schizothecium* based on ribosomal DNA and protein coding genes. Fungal Divers..

[37] Lu Y.Z., Boonmee S., Dai D.Q., Liu J.K., Hyde K.D., Bhat D.J., Ariyawansa H., Kang J.C. (2017). Four new species of *Tubeufia* (Tubeufiaceae, Tubeufiales) from Thailand. Mycol. Prog..

[38] Dissanayake A.J., Bhunjun C.S., Maharachchikumbura S.S.N., Liu J.K. (2020). Applied aspects of methods to infer phylogenetic relationships amongst fungi. Mycosphere.

[39] Katoh K., Rozewicki J., Yamada K.D. (2019). MAFFT online service: Multiple sequence alignment, interactive sequence choice and visualization. Brief. Bioinform..

[40] Capella-Gutiérrez S., Silla-Martínez J.M., Gabaldón T. (2009). TrimAl: A tool for automated alignment trimming in large-scale phylogenetic analyses. Bioinformatics.

[41] Stamatakis A., Hoover P., Rougemont J. (2008). A rapid bootstrap algorithm for the RAxML web servers. Syst. Biol..

[42] Miller M.A., Pfeiffer W., Schwartz T. Creating the CIPRES Science Gateway for inference of large phylogenetic trees. Proceedings of the 2010 Gateway Computing Environments Workshop (GCE).

[43] Hyde K.D., Hongsanan S., Jeewon R., Bhat D.J., McKenzie E.H.C., Jones E.B.G., Phookamsak R., Ariyawansa H.A., Boonmee S., Zhao Q. (2016). Fungal diversity notes 367–490: Taxonomic and phylogenetic contributions to fungal taxa. Fungal Divers..

[44] Corda A.K.J. (1837). Incones Fungorum Hucusque Cognitorum.

[45] Linder D.H. (1929). A monograph of the helicosporous fungi imperfecti. Ann. Mo. Bot. Gard..

[46] Goos R.D. (1986). A review of the anamorph genus *Helicoma*. Mycologia.

[47] Li W.J., McKenzie E.H.C., Liu J.K., Bhat D.J., Dai D.Q., Camporesi E., Tian Q., Maharachchikumbura S.S.N., Luo Z.L., Shang Q.J. (2020). Taxonomy and phylogeny of hyaline-spored coelomycetes. Fungal Divers..

[48] Lu Y.Z., Liu J.K., Hyde K.D. (2020). Proposal to conserve Pseudohelicomyces YZ Lu & al. (Tubeufiaceae) against *Pseudohelicomyces* Garnica & E. Valenz. (Hymenogastraceae). Taxon.

[49] Hsieh S.Y., Goh T.K., Kuo C.H. (2021). New species and records of Helicosporium sensu lato from Taiwan, with a reflection on current generic circumscription. Mycol. Prog..

[50] Rossman A.Y. (1977). The genus *Ophionectria* (Euascomycetes, Hypocreales). Mycologia.

[51] Rao P.R., Rao D. (1964). Some helicosporae from Hyderabad-II. Mycopathol. Mycol. Appl..

[52] Penzig O.A.J., Saccardo P.A. (1897). Diagnoses fungorum novorum in Insula Java collectorum. Series secunda. Malpighia.

[53] Luo Z.L., Bhat D.J., Jeewon R., Boonmee S., Bao D.F., Zhao Y.C., Chai H.M., Su H.Y., Su X.J., Hyde K.D. (2017). Molecular phylogeny and morphological characterization of asexual fungi (Tubeufiaceae) from freshwater habitats in Yunnan, China. Cryptogam. Mycol..

[54] Seaver F.J. (1909). The Hypocreales of north America–I. Mycologia.

[55] Seaver F.J., Waterston J.M. (1940). Contributions to the mycoflora of Bermuda–I. Mycologia.

[56] Zhao G.Z., Liu X.Z., Wu W.P. (2007). Helicosporous hyphomycetes from China. Fungal Divers..

[57] Boonmee S., Wanasinghe D.N., Calabon M.S., Huanraluek N., Chandrasiri S.K., Jones G.E., Rossi W., Leonardi M., Singh S.K., Rana S. (2021). Fungal diversity notes 1387–1511: Taxonomic and phylogenetic contributions on genera and species of fungal taxa. Fungal Divers..

[58] Dayarathne M.C., Maharachchikumbura S.S.N., Jones E.B.G., Dong W., Devadatha B., Yang J., Ekanayaka A.H., De Silva W., Sarma V.V., Al-Sadi A.M. (2019). Phylogenetic revision of Savoryellaceae and evidence for its ranking as a subclass. Front. Microbiol..

[59] Chethana K.W.T., Manawasinghe I.S., Hurdeal V.G., Bhunjun C.S., Appadoo M.A., Gentekaki E., Raspé O., Promputtha I., Hyde K.D. (2021). What are fungal species and how to delineate them. Fungal Divers..

[60] Pem D., Jeewon R., Chethana K.W.T., Hongsanan S., Doilom M., Suwannarach N., Hyde K.D. (2021). Species concepts of Dothideomycetes: Classification, phylogenetic inconsistencies and taxonomic standardization. Fungal Divers..

[61] Hyde K.D., Jeewon R., Chen Y.J., Bhunju C.S., Calabon M.S., Jiang H.B., Lin C.G., Norphanphoun C., Sysouphanthong P., Pem D. (2020). The numbers of fungi: Is the descriptive curve flattening?. Fungal Divers..

